# Glutamate mediated metabolic neutralization mitigates propionate toxicity in intracellular *Mycobacterium tuberculosis*

**DOI:** 10.1038/s41598-018-26950-z

**Published:** 2018-05-31

**Authors:** Jae Jin Lee, Juhyeon Lim, Shengjia Gao, Christopher P. Lawson, Mark Odell, Saki Raheem, JeongIm Woo, Sung-Ho Kang, Shin-Seok Kang, Bo-Young Jeon, Hyungjin Eoh

**Affiliations:** 1 Department of Molecular Microbiology and Immunology, Keck School of Medicine, University of Southern California, Los Angeles, CA 90033 USA; 2 Strathclyde Institute of Pharmacy and Biomedical Sciences, University of Strathclyde, Glasgow, G4 0RE United Kingdom; 3 School of Life Sciences, University of Lincoln, Brayford Pool, Lincoln, LN6 7TS United Kingdom; 4 Department of Life Sciences, Faculty of Science and Technology, University of Westminster, W1W 6UV London, United Kingdom; 5 Department of Biomedical Laboratory Science, College of Health Science, Yonsei University, Wonju, 26493 Korea; 6 Chungbuk Veterinary Service Laboratory, 380-230 Chungju, Republic of Korea

## Abstract

Metabolic networks in biological systems are interconnected, such that malfunctioning parts can be corrected by other parts within the network, a process termed adaptive metabolism. Unlike Bacillus Calmette-Guérin (BCG), *Mycobacterium tuberculosis* (Mtb) better manages its intracellular lifestyle by executing adaptive metabolism. Here, we used metabolomics and identified glutamate synthase (GltB/D) that converts glutamine to glutamate (Q → E) as a metabolic effort used to neutralize cytoplasmic pH that is acidified while consuming host propionate carbon through the methylcitrate cycle (MCC). Methylisocitrate lyase, the last step of the MCC, is intrinsically downregulated in BCG, leading to obstruction of carbon flux toward central carbon metabolism, accumulation of MCC intermediates, and interference with GltB/D mediated neutralizing activity against propionate toxicity. Indeed, vitamin B12 mediated bypass MCC and additional supplement of glutamate led to selectively correct the phenotypic attenuation in BCG and restore the adaptive capacity of BCG to the similar level of Mtb phenotype. Collectively, a defective crosstalk between MCC and Q → E contributes to attenuation of intracellular BCG. Furthermore, GltB/D inhibition enhances the level of propionate toxicity in Mtb. Thus, these findings revealed a new adaptive metabolism and propose GltB/D as a synergistic target to improve the antimicrobial outcomes of MCC inhibition in Mtb.

## Introduction

*Mycobacterium tuberculosis* (Mtb), the etiological agent of tuberculosis (TB), claimed 1.8 million human lives in 2016, the highest cause of human mortality among infectious diseases including AIDS^1^. Chemotherapies developed around 50 years ago rendered TB initially curable, but the incidence has resurged due to the emergence of multidrug-resistant (MDR)-TB strains that are virtually untreatable with standard TB treatment. The urgent need to develop new anti-infectives against drug-resistant TB has been compounded by the threat of extensively drug-resistant (XDR)^2^ and totally drug-resistant (TDR) TB strains^3^. Drug-resistance in Mtb is a serious concern that will undoubtedly worsen the global TB pandemic. Unfortunately, the spread of drug-resistance in Mtb has outpaced the introduction of new drugs.

Despite the inextricable link between uncontrolled TB incidence and intracellular adaptation, Mtb appears to lack classical virulent factors such as specialized modulators that interact with the host immune system^4^. Instead, Mtb has evolved to undergo adaptive metabolism by altering its lifestyle to ensure persistence in the host, and to maximize tolerance against host imposed stresses including immune response^5^. As such, adaptive metabolism to nutritionally variable, but incompletely defined, niches inside hosts is a hallmark that Mtb uses for species conservation. When entering the host environments, Mtb continuously switches its cellular compartments of residence and acquires anabolic sources from the host while evading the immune system mediated inflammation. Mtb stimulates the import and subsequent degradation of intracellular storage molecules into compounds that can be catabolized. For this, Mtb remodels its metabolic networks in order to assimilate new nutrients and, more importantly, to neutralize any adverse consequences that may occur during catalysis, processes collectively termed adaptive metabolism^6–10^. This metabolic feature allows for maximum efficiency in the utilization of host nutrients and optimal virulence^11^. Such metabolic plasticity coupled with the ineffective standard TB treatment strategy renders Mtb one of the most successful pathogens.

Bacillus Calmette-Guérin (BCG), the only approved TB vaccine, was developed from virulent *Mycobacterium bovis* by consecutive subculture for 12 years on nutrient enriched media, during which the mother *M*. *bovis* strain lost its virulence due to an inadequately-defined manufacturing process^12^. Consequently, BCG uses the host imposed carbon substrates inefficiently and fails to manage its intracellular lifestyle within host environments. Although many studies have attempted to reveal the factors that caused its attenuation and sought to apply these findings pharmacologically^13,14^, there is still no clear explanation. Comparative genomics of Mtb and BCG identified multiple truncations in genetic regions in BCG including region of difference (RD) 1 locus, 506 synonymous and 769 nonsynomynous SNPs compared with Mtb H_37_Rv strain^15^, tandem duplications of DU1 and DU2^16^. Among the intensive genetic modifications, truncation of RD-1 is observed across all BCG strains and RD-1 locus is shown to encode mediators promoting Mtb pathogenesis^17–20^. The RD1 locus of Mtb includes early secreted antigenic target (ESAT-6), secretion system-1 (ESX-1), and culture filtrate protein (CFP-10), all of which were known to play significant roles in Mtb virulence and interaction with host macrophages^21–24^. However, these genetic defects in BCG have yet to fully explain its phenotypic attenuation in the host^14,25^.

Recent studies have shown that Mtb has evolved to optimize specific metabolic activities that enable the use of multiple carbon sources simultaneously, thereby increasing its ability to survive in a nutritionally adverse environment^26,27^. In the present study, we hypothesized that BCG lacks the adaptive metabolic plasticity needed to efficiently consume host nutrients; this metabolic defect in BCG would allow it to be exploited to reveal conceptually novel anti-TB drug targets. We used metabolomics profiling of Mtb and BCG and identified Mtb-unique adaptive strategies. It includes the methylcitrate cycle (MCC) as an assimilation route of host carbons such as propionate, and the glutamine (Q)-glutamate (E) converting (Q → E) activity by glutamate synthase (GltB/D) as a neutralization effort to mitigate propionate toxicity while using MCC. Efficient coordination of the interaction between MCC and Q → E activity is an essential component for Mtb’s intracellular lifestyle, this functional crosstalk is relatively inefficient in BCG metabolism that may contribute to intracellular phenotypic attenuation. The discovery of functional interaction between MCC and Q → E identifies GltB/D as a new potential drug target whose inhibition mimics the bactericidal properties of Mtb’s MCC activity by maximizing the propionate toxicity.

## Results

### BCG has a narrower carbon source preference for *in vitro* growth

Unlike H_37_Rv wild type *M*. *tuberculosis* (WT Mtb), whose *in vitro* growth is supported by a wide range of carbon species^28^, BCG (Pasteur 1173P2) has a much narrower carbon source preference to support its *in vitro* growth. Among the carbon species tested in the present study, *in vitro* BCG growth was greatest under 0.2% glycerol while there was no recognizable growth under either 0.2% propionate or 0.2% dextrose as a single source of carbon. However, there was intermediate growth under 0.2% acetate (Fig. S1A). Full growth of BCG under 0.2% glycerol was gradually suppressed by the presence of increasing amounts of propionate and completely suppressed at or above 0.2% propionate (Fig. S1B), whereas these are all growth-permissive conditions for WT Mtb (Fig. S1C). Propionate-mediated growth suppression in BCG was associated with ~1 log_10_ reduction in viability, with a more severe reduction (~2 log_10_) in the presence of dual carbons (glycerol and propionate) over a one-week incubation (Fig. S1D). Different BCG strains such as Swedish strain (TMC 1009) (Fig. S2A) and Danish strain (TMC 1010) (Fig. S2B) also exhibit similar carbon source preference, indicating a common metabolic feature that is associated with *in vitro* BCG phenotype. These results suggest that propionate metabolism in BCG is less efficient than that of WT Mtb and subsequent propionate toxicity severely attenuates BCG growth *in vitro*.

### Metabolic profiling of BCG to define propionate toxicity

**Figure 1:**
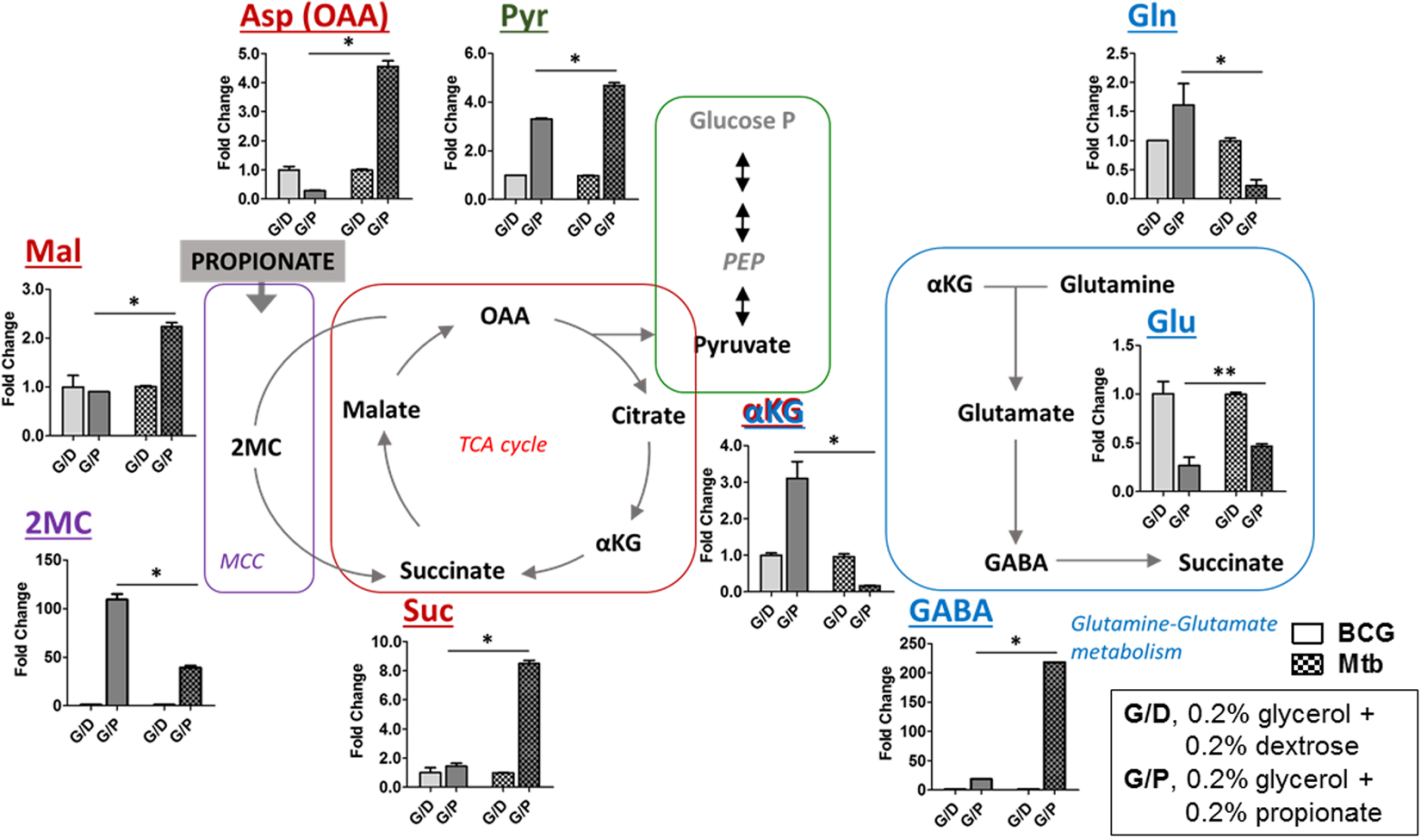
Carbon source dependent metabolic consequences in WT Mtb and BCG. Intrabacterial pool sizes of metabolic intermediates in the TCA cycle (red box), methylcitrate cycle (purple box), glycolysis (green box), and nitrogen metabolism (blue box) in BCG (left half in each bar graph) and WT Mtb (right half in each graph) incubated in media with either 0.2% glycerol and 0.2% dextrose (G/D, light gray) or 0.2% glycerol and 0.2% propionate (G/P, dark gray) carbons for 24 hrs are depicted. Total bar heights indicate the intrabacterial pool sizes relative to their counterparts in BCG and WT Mtb cultured in G/D. All values are the average of biological triplicates ± s.e.m. *P < 0.001; **P < 0.01 by Student’s unpaired t-test. αKG, α-ketoglutarate; Suc, succinate; Mal, malate; Asp, aspartate; OAA, oxaloacetate; 2MC, 2-methylcitrate; Pyr, pyruvate; Glu, glutamate; Gln, glutamine; GABA, γ-aminobutyric acid.

To elucidate the metabolic vulnerability of BCG in its inability to adapt to propionate carbon source, we conducted liquid chromatography mass spectrometry (LC-MS) metabolomics of BCG and WT Mtb following exposure to “glycerol and dextrose” (G/D) or “glycerol and propionate” (G/P) medium (Fig. S1C). We then sampled the metabolome before a measurable loss of viability. Focusing on **I** carbon (fatty acid) metabolism such as the methylcitrate cycle (MCC) (purple box) and the TCA cycle (red box) and **II** nitrogen metabolism such as glutamine (Q)-glutamate (E) pathway and GABA shunt (blue box), we observed the following specific metabolic defects in BCG (Fig. 1).

#### MCC and TCA cycle

There was a specific and time-dependent accumulation of MCC intermediates such as 2-methylcitrate (2MC) and 2-methylisocitrate (2MI) in both BCG and WT Mtb under G/P carbons, but not under G/D carbons (Fig. 1; purple box), likely due to activated propionate consumption through MCC^29,30^. Although there were seemingly similar consequences for both strains, exposure to the same amount of G/P carbons reproducibly induced around 3~4 times greater accumulation of 2MC and 2MI in BCG than in WT Mtb (Figs 1 and S3A–C). In BCG, the accumulation of carbon substrates in the form of MCC intermediates derived from the G/P carbons slowed the biosynthesis of the reductive branch of TCA cycle intermediates (succinate, malate and aspartate) as compared to that of WT Mtb (Fig. 1; red box).

#### Glutamine (Q)-Glutamate (E) pathway and GABA shunt

In BCG under G/P carbons, we also observed that pool sizes of alpha-ketoglutarate (αKG) and glutamine (Q) were upregulated with a reciprocal depletion of glutamate (E) as compared to those under G/D carbons; this occurred in the opposite direction in WT Mtb (Fig. 1; blue box), suggesting that the BCG specific metabolic vulnerability includes a dysregulation of glutamine-glutamate conversion (Q → E). Indeed, the E to Q conversion ratio (E/Q) in BCG under G/P carbons was ~0.17 while it was ~1.85 under G/D carbons. In contrast, the E/Q value in WT Mtb was relatively unaffected by the carbon conditions: ~4.04 under G/D and ~4.26 under G/P carbons (Fig. S3D,E), implying a functional link of Q → E activity to propionate assimilation through MCC activity. Decreased Q → E activity (i.e., lower value of E/Q) in BCG also limited the carbon flow towards the GABA shunt. These metabolomics profiles of BCG and WT Mtb imply that metabolic activities to conserve the glutamate pool are functionally associated with the efficient catalysis of G/P carbons in WT Mtb, a process that is relatively weak in BCG metabolism (Fig. S3E).

#### Irrelevance to RD1 truncation

To examine whether the propionate toxicity and dysregulated Q-E pathway in BCG metabolism under G/P carbons is due to loss of the RD1 locus, we generated an RD1 depleted *M*. *bovis* strain (ΔRD1) by a recombineering technology as was previously applied to generate ΔRD1 in WT Mtb^31^. The RD1 truncation in *M*. *bovis* was confirmed by whole genome sequencing (ChunLab, Inc., Seoul National University, South Korea) and multiplex PCR analyses using validated PCR primer sets (Fig. S4A and Table S1)^32^. Subsequently, we conducted LC-MS metabolomics of WT *M*. *bovis* and ΔRD1. Metabolomic alterations in fatty acid metabolism (MCC and TCA cycle) and glutamine (Q)-glutamate (E) metabolism of *M*. *bovis* following exposure to G/P carbons resemble those of WT Mtb under G/P carbons (Figs 1 and S4B). G/P carbons induced *M*. *bovis* MCC activity as evidenced by vigorous accumulation of 2MC/2MI pools by ~15 folds compared to those under G/D carbons. These MCC intermediates efficiently serve the substrates for the biosynthesis of the TCA cycle intermediates such as succinate, malate, and aspartate. By calculating the E/Q values, we also observed greater Q to E conversion activity of *M*. *bovis* (~9.4 under G/D and ~19.3 under G/P) (Fig. S4B) compared to those of WT Mtb to secure the intrabacterial glutamate pool, serving the substrate for GABA biosynthesis. Intriguingly, there were no major metabolic perturbations in intermediates involved in fatty acid metabolism, the Q-E pathway, and the GABA shunt in ΔRD1 as compared to those in *M*. *bovis* (Fig. S4B). Furthermore, both *M*. *bovis* and ΔRD1 strains share the preferred carbon species for *in vitro* growth. Interestingly, G/P carbons supported the *in vitro* growth of both strains better than G/D carbons did at later stages (Fig. S4C), presumably due to the greater MCC and E → Q activities (Fig. S1C). Based on the foregoing metabolomics profiles and *in vitro* growth analyses, we inferred that the metabolic defect in BCG under G/P carbons is not simply due to disruption of RD1; rather due to the loss of adaptive metabolism.

In summary, WT Mtb metabolism required for the efficient utilization of G/P carbons comprises MCC mediated propionate assimilation and functionally linked Q-E pathway (Fig. 1), that are significantly weaker in BCG metabolism. Furthermore, the metabolic vulnerability of BCG in response to G/P carbons is not related with an RD1 genetic deficiency.

### Intracellular BCG and Mtb elicit distinct MCC activities

**Figure 2:**
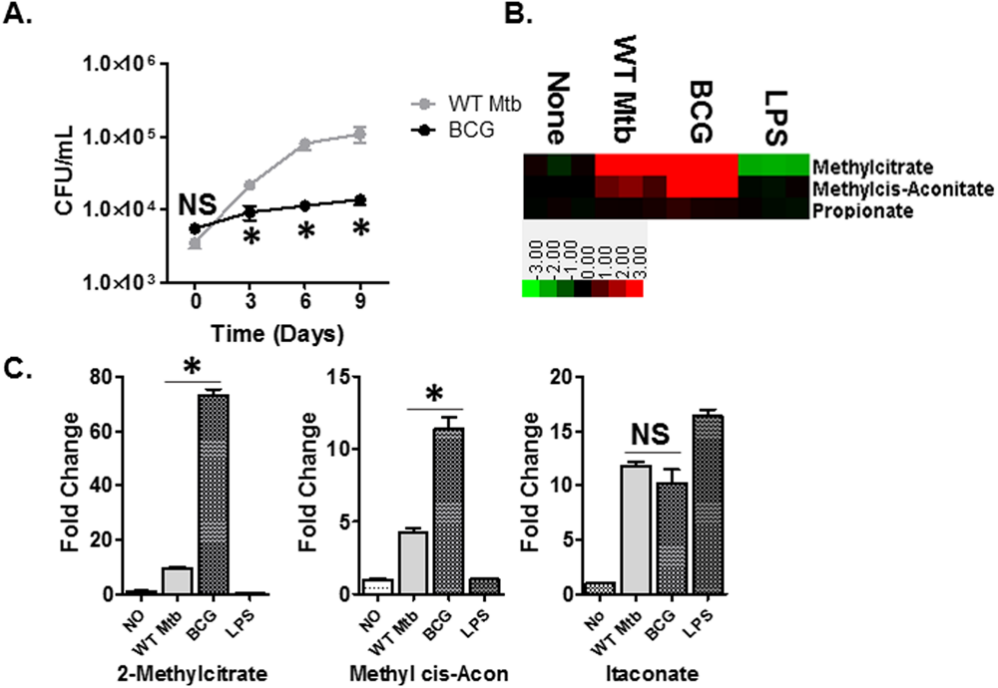
Phenotypic and metabolic states of WT Mtb and BCG inside THP-1 macrophages. (**A**) CFU-based viability test of WT Mtb and BCG inside THP-1 over 9 days of incubation. *P < 0.001 by Student’s unpaired t-test. (**B**) Heatmap profile of THP-1 following either infection with WT Mtb or BCG at MOI 1.0 or treatment of 10 µg/mL LPS. Columns depict experimental conditions, starting with untreated control followed by WT Mtb, BCG infection, and LPS treatment. Rows indicate metabolites of the bacterial propionate pathway (methylcitrate cycle, MCC). Data are depicted on a log_2_ scale relative to untreated control for each experimental condition. (**C**) Fold changes of MCC intermediates relative to those in uninfected control. Change of itaconate was included as a control in THP-1 activation. *P < 0.001; and N.S, not significant by Student’s unpaired t-test.

BCG showed slowed intracellular replication compared to that of WT Mtb (Fig. 2A). To elucidate if we could find evidence of propionate toxicity as a contributing factor to its attenuated adaptation to host nutrient environment, we conducted metabolomics profiles of THP-1 human macrophages (THP-1) that were infected with BCG or WT Mtb. Unsupervised hierarchical clustering analyses of total metabolomes identified metabolite subsets that were both common and unique to each condition (Fig. S5A). The metabolomics profiles primarily identified core metabolic processes common to all conditions (Table S2), including downregulation of intermediates in the TCA cycle (as a readout of impaired respiration) with reciprocal accumulation in succinate and itaconate, a systemic metabolic signature of the host’s immunemetabolic activation commonly seen following microbial infection and LPS treatment (Figs 2C, S5A and Table S2).

Given our findings on *in vitro* metabolomics profiles and growth of BCG, we directed specific attention to propionate metabolism. As shown in Fig. 2B, we observed an upregulation in intermediates of bacterial MCC, including 2MC, 2MI, and 2-methyl cis-aconitate (2MA) in THP-1 infected with BCG or WT Mtb, but not in THP-1 treated with LPS. To confirm their origin, we sampled the THP-1 metabolome after removing the un-lysed infecting bacilli^33^. The Metabolomics profile of THP-1 free of infected bacilli lost the chromatograms of 2MC/2MI and 2MA, but retained that of itaconate (Fig. S5B) which suggested that they are metabolites of infecting bacilli. We also noted that the relative abundance of 2MC/2MI and 2MA were significantly greater in THP-1 infected with BCG than WT Mtb by 7.7 and 2.8 times, respectively (Fig. 2C). There was no significant difference in itaconate, consistent with the *in vitro* metabolomics profiles of BCG and WT Mtb under G/P carbons (Figs 1 and S3D–F). The results indicate that **I** the carbons of the THP-1 cell compartments where WT Mtb or BCG resides stimulate MCC activity, **II** intracellular BCG and WT Mtb elicit distinct MCC activities, which are **III** the potential contributors to the attenuated replication of intracellular BCG.

### Mtb maintains lower MCC intermediates by ICL activity under G/P carbons

**Figure 3:**
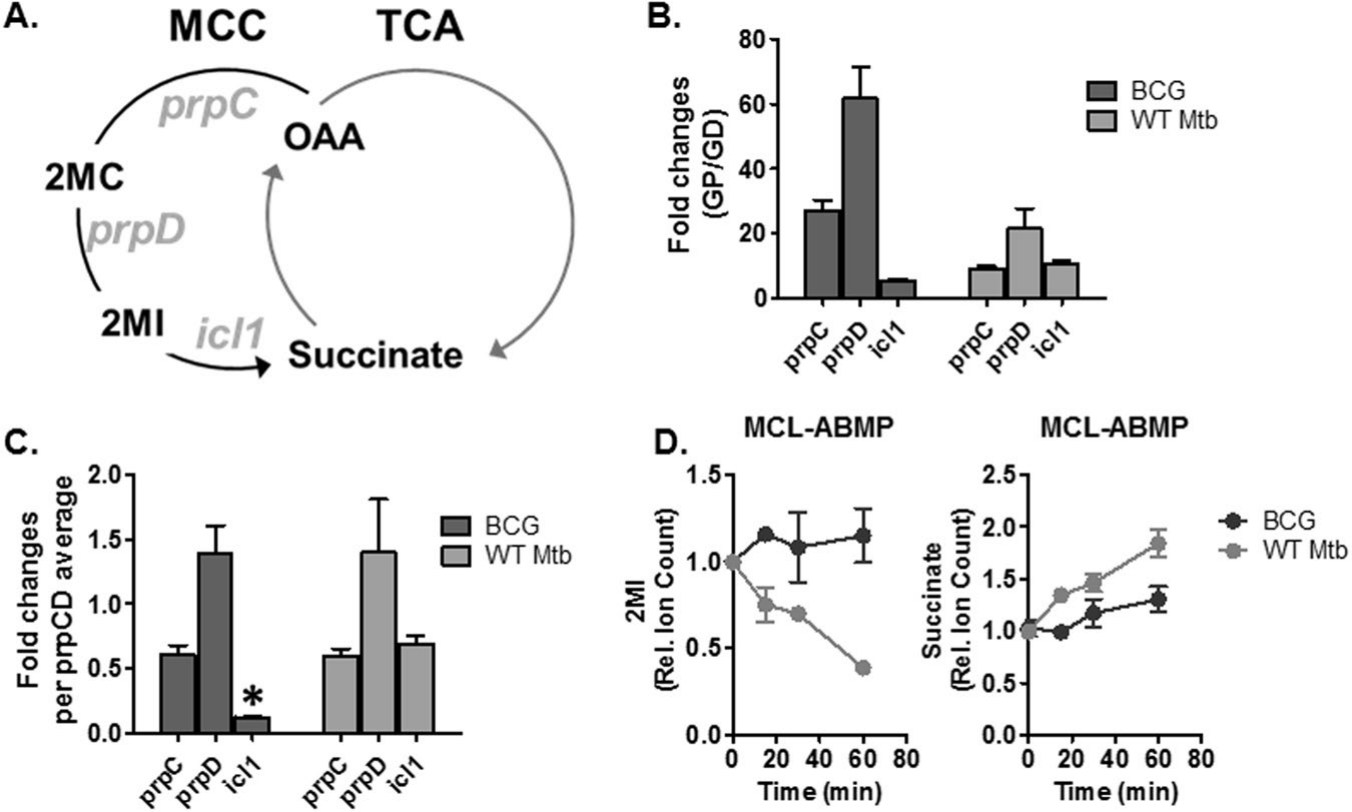
Intrinsic downregulation of methylisocitrate lyase activity causes toxic MCC intermediates in BCG. (**A**) The schematic describes the genes and intermediates in the bacterial methylcitrate cycle, MCC. (**B**,**C**) Expression levels of transcripts of *prpC*, *prpD* and *icl1* in WT Mtb or BCG cultured under G/P carbons relative to their counterparts under G/D carbons are described (**B**) and their changes are demonstrated by fold change relative to the average of the first two genes (*prpC* and *prpD*). Asterisk indicates the relative level of BCG *icl1* transcript as the lowest (**C**). (**D**) MCL-ABMP mediated time-course measurements of MCL substrate (2MC/2MI) and product (succinate) in the presence of either WT Mtb or BCG small metabolome extract (SME) under G/P carbons. All values are average of two independent experiments, each of which consisted of biological triplicates ± SD.

It was intriguing that the levels of accumulation in MCC intermediates between WT Mtb and BCG at all conditions tested were different (Figs 1, 2C, S3A–C and S5B). To clarify the mechanistic bases, we first monitored the levels of mRNA expression of MCC genes *prpC*, *prpD*, and *icl1* (Fig. 3A). As expected, mRNA transcripts of all MCC components in both WT Mtb and BCG under G/P carbons were drastically induced compared to those under G/D carbons (Fig. 3B). If the level of *icl1* transcript is indicated by the fold change relative to the average level of *prpC* and *prpD*, *icl1* in BCG was ~0.12, while it was ~0.72 in WT Mtb, inferring that the relative induction of *icl1* in BCG under G/P carbons was significantly lower (Fig. 3C). To further correlate *icl1* mRNA change with catalytic activity, as a last step of MCC (methylisocitrate lyase, MCL), we conducted activity based metabolomics profiling (ABMP)^34^. The ABMP reactions included purified recombinant ICL1, divalent cation (Mg^2+^), and small metabolite extracts (SME) of either WT Mtb or BCG grown under G/P carbons. ICL1 (as an MCL enzyme)-mediated changes of pool sizes in SME metabolites were monitored by LC-MS. A time course MCL-ABMP allowed us to trace the changes in abundance of 2MI and succinate as substrate and product of MCL, respectively (Fig. 3A). The abundance of 2MI in an ABMP reaction with WT Mtb SME was significantly decreased by up to ~75% in 1 hr incubation at 37 °C, with only a minor change in 2MI abundance in an ABMP reaction with BCG SME. Reciprocally, an ABMP reaction with WT Mtb SME showed an increase in abundance of succinate (an MCL product), as compared to the ABMP reaction with BCG SME (Fig. 3D). ABMP reaction with no ICL1 was used as a negative control. The foregoing qrtRCR and MCL-ABMP assays showed that the catalytic activity of BCG ICL1 was transcriptionally and catalytically downregulated in adapting to the use of G/P carbons. Consequently, toxic MCC intermediates were ~4 times greater in growth-nonpermissive BCG.

### Propionate toxicity acidifies the BCG cytoplasm

**Figure 4:**
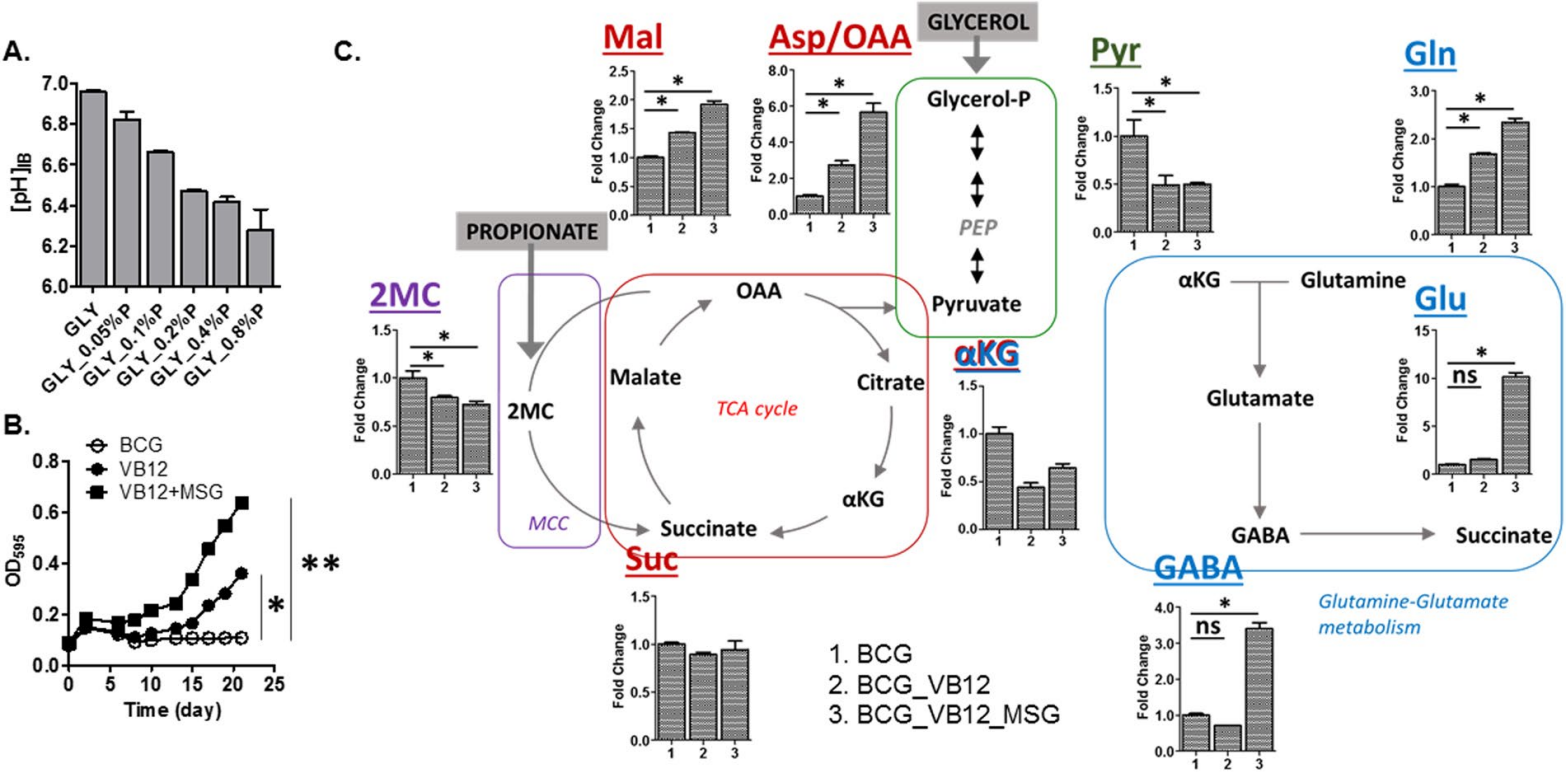
Chemical and genetic rescue of growth-nonpermissive BCG under G/P carbons. (**A**) Determination of intrabacterial pH of BCG cultured in the presence of 0.2% glycerol and various concentrations of propionate (0.05 to 0.8%) for 24 hrs. (**B**) Effect of cyanocobalamin (VB12) and/or monosodium glutamate (MSG) on CFU-based viability of BCG cultured under G/P carbons. *P < 0.01; **P < 0.001 by Student’s unpaired t-test. All values are the average of two independent experiments ± SD, each of which consisted of biological triplicates. (**C**) Metabolic consequences of VB12 and/or MSG supplement on BCG metabolomics profiles; intermediates of TCA cycle (red box), MCC (purple box), glycolysis (green box), and nitrogen metabolism (blue box) cultured under G/P carbons. Bar heights indicate the relative extent of intrabacterial concentration of intermediates. *P < 0.01; ns, not significant by Student’s unpaired t-test. All values are the average of two independent experiments ± s.e.m, each of which consisted of biological triplicates. αKG, α-ketoglutarate; Suc, succinate; Mal, malate; Asp, aspartate; OAA, oxaloacetate; 2MC, 2-methylcitrate; Pyr, pyruvate; Glu, glutamate; Gln, glutamine; GABA, γ-aminobutyric acid.

Metabolomics profiles, *in vitro* growth, and viability analyses have indicated that metabolic defects of BCG under G/P carbons are similar to those of ICL deficient Mtb (ICL KO)^29^. To prove that BCG displays an analogous metabolic perturbation to ICL KO in response to G/P carbons, we monitored the cytoplasmic pH of BCG using a pH sensitive GFP construct^29,35^. Exposure of BCG to G/P carbons rapidly acidified the cytoplasmic pH from ~7.0 to ~6.3 in a time- and propionate concentration- dependent manner (Fig. 4A); this was not seen in WT Mtb under G/P carbons or in BCG under G/D carbons^29^. A previous study showed that cyanocobalamin (vitamin B12, VB12) can activate an alternate propionate metabolism, the methylmalonyl-CoA pathway^36,37^, subsequently protecting ICL KO from bactericidal effects due to propionate toxicity^29^. Thus, we tested the ability of VB12 to rescue BCG growth under G/P carbons. Indeed, BCG growth was partially restored by VB12 treatment (Fig. 4B), which was preceded by metabolic restoration of intermediates in both MCC (2MC and 2MI) and the TCA cycle (aspartate and malate) (Fig. 4C). These results identified that defective MCL activity following accumulation of toxic MCC intermediates, and cytoplasmic acidification are the major metabolic causes of impaired BCG adaptation to propionate carbon and the intracellular nutrient environment.

### Q-E conversion activity buffers cytoplasmic pH during using MCC

BCG remains viable under acidic environment by maintaining cytoplasmic pH within a physiological range (Fig. S6A)^38^. There is substantial evidence suggesting the essential role of central nitrogen metabolism in adapting to an acidic environment, which includes glutamate dehydrogenase and glutamine-αKG aminotransferase in Q-E pathway, or glutamate decarboxylase in GABA shunt^39–43^. Here, we observed that loss of BCG viability upon exposure to G/P carbons is at least in part due to cytoplasmic acidification (Figs 1, 4A and S1D), thus inferring that propionate toxicity interferes with adaptive metabolism required for adjustment of cytoplasmic pH. A metabolomics profile of BCG under an acidic environment (Fig. S6B,C) had a similar E/Q to that of BCG under G/D (growth-permissive), but this value was significantly greater than the level in BCG under G/P (growth-nonpermissive) (Fig. S3D–F). This indicates that catalytic activity involved in Q → E is significantly downregulated in BCG during assimilating propionate carbons through MCC.

Based upon the metabolomics profiles of BCG under G/P carbons and in an acidic environment, we hypothesized that BCG succumbed while adapting to G/P carbons due to direct damage — accumulation of MCC intermediates/propionate toxicity. In addition, we believe that the bactericidal effects are indirectly exacerbated by defective Q → E activity. To assess the role of Q → E activity in mitigating propionate toxicity, we tested the effects of monosodium glutamate (MSG, cell permeable glutamate) as a product of glutamate synthase (GltB/D, involved in Q → E) or glutamine as a substrate of GltB/D on BCG growth. We observed that MSG, not glutamine, further improved the VB12-mediated rescue of BCG growth (Fig. 4B) to a level similar to that of WT Mtb (Fig. S1C). Chemically (MSG and VB12) rescued BCG growth was also preceded by the restoration of not only metabolomics profiles including glutamate and GABA pool sizes but also cytoplasmic pH (Fig. 4C), confirming the crucial role of GltB/D as a Q → E activity and following GABA biosynthesis as metabolic efforts to neutralize propionate toxicity under active MCC.

Next, to assess the catalytic link between GltB/D that mediates Q → E and MCC activities (Fig. S6D), we monitored the effects of accumulated MCC intermediates on the GltB/D activity by an *in vitro* GltB/D enzyme reaction. To monitor the GltB/D activity, we added αKG and glutamine as GltB/D’s substrates and BCG lysates prepared from the G/D culture or G/P culture as a source of GltB/D enzyme (Fig. S6D). Then, the production of glutamate or consumption of substrates were monitored by LC-MS after 10 min incubation at 37 °C. Greater Q → E activity was observed when the reaction contains BCG lysate of G/D culture compared to that of BCG lysate of G/P culture (Fig. S6D). In a separate reaction, the GltB/D activity in WT Mtb lysate of G/D culture was significantly downregulated when the reaction included chemically synthesized 2MI (Fig. S7), one of MCC intermediates and direct substrate of ICL’s MCL activity, proving the interactive crosstalk between MCC activity and GltB/D mediated Q → E activity while catabolizing G/P carbons (Fig. S6D).

**Figure 5:**
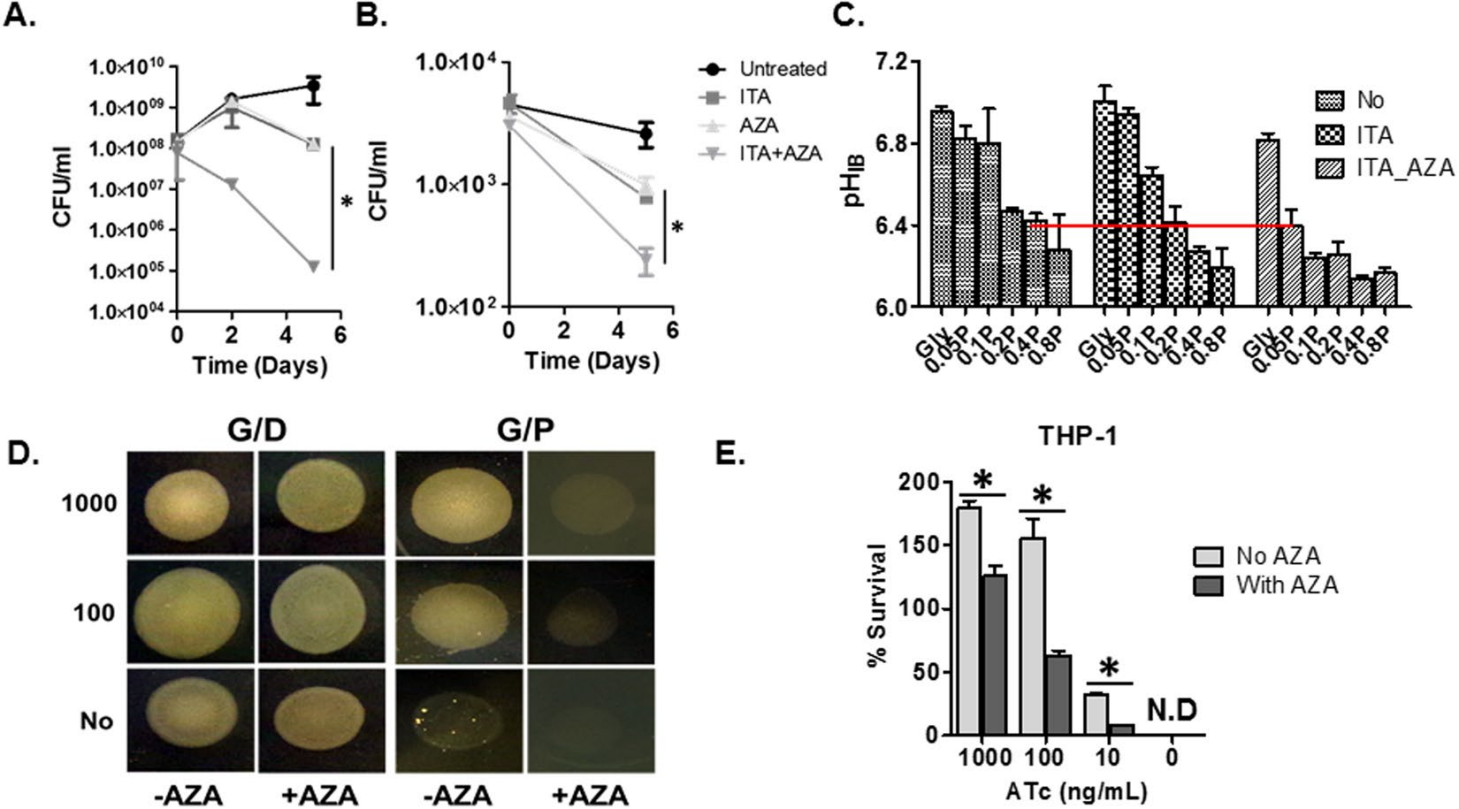
GltB/D mediated Q-E conversion activity dampens propionate toxicity. (**A**,**B**) The effects of chemical (itaconate and azaserine) inactivation on CFU-based BCG viability *in vitro* culture containing 0.2% glycerol and 0.1% propionate (**A**) and in THP-1. (**B**) *P < 0.005 by ANOVA with Bonferroni post-test correction. (**C**) Biochemical consequences of co-treatment of both itaconate and Azaserine were measured by intrabacterial pH of BCG in media containing 0.2% glycerol with varying concentrations of propionate (0.05 P, 0.05% propionate; 0.1 P, 0.05% propionate; 0.2 P, 0.2% propionate; 0.4 P, 0.4% propionate; and 0.8 P, 0.8% propionate). Red line indicates the conditions whereat intrabacterial pH is pH 6.4. All values are the average of two independent experiments ± SD, each of which consisted of biological triplicates. (**D**) Colony formation of ICL KD under varying doses of ATc (0~1000 ng/mL) in the presence or absence of 1.0 µg/mL Azaserine under either G/D or G/P carbons. (**E**) CFU-based ICL KD viability in THP-1 (**E**). The values (**A**,**B**,**D** and **E**) are the average of two independent experiments ± s.e.m, each of which consisted of biological triplicates. *P < 0.001 by Student’s unpaired t-test. ITA, itaconate; AZA, Azaserine.

In addition to MSG mediated BCG rescue and *in vitro* 2MI mediated inhibition against GltB/D activity, we also showed a functional role of Q → E in buffering propionate toxicity through the use of an GltB/D inhibitor (Azaserine, AZA) (Fig. S8A)^44^. Treatment with AZA to WT Mtb led to an accumulation of αKG and reciprocal depletion of glutamate, suggesting that under the conditions tested in this study, the dominant AZA activity in intact WT Mtb is a GltB/D inhibitor (Fig. S8B) and this was corroborated by separate finding that AZA inhibits the production of glutamate in an *in vitro* GltB/D reaction as performed to test the 2MI inhibitory effect (Fig. S6D). A single treatment of an ICL inhibitor (itaconate, ITA) inhibited *in vitro* BCG growth, with no prominent loss of viability under 0.2% glycerol and 0.1% propionate. However, co-treatment with both ITA and AZA rapidly killed BCG by ~3 log_10_ over 5-day incubation, with a drastic cytoplasmic acidification (pH ~6.3) equivalent to the pH when BCG was exposed to 0.2% glycerol and 0.8% or greater propionate concentrations (Figs 5A,C and S1D). A similar inhibitory effect of ITA and AZA on intracellular BCG viability was also observed within THP-1 (Fig. 5B). The MSG rescue and GltB/D inhibition studies confirm that Q → E is a metabolic neutralization effort to dampen propionate toxicity by pH buffering during assimilation of propionate through MCC. This provides evidence that crosstalk between Q → E and MCC is one example of adaptive metabolism in WT Mtb under G/P carbons.

### Inactivation of Q → E exacerbates propionate toxicity in ICL deficient Mtb

The metabolic defects in BCG under G/P carbons revealed an unprecedented role of Q → E activity in mitigating propionate toxicity as part of adaptive metabolism, an essential component for the Mtb intracellular lifestyle. Thus, we hypothesized that disruption of the crosstalk between Q → E and MCC exacerbates propionate toxicity in WT Mtb such that it is similar to BCG metabolism, thereby interfering with intracellular Mtb adaptation. To confirm that functional interaction between Q → E and MCC can serve as a potential TB drug target, we used a tetracycline inducible conditional knockdown strain of Mtb ICL (ICL KD)^45^ and identified that the *in vitro* growth of ICL KD under 0.2% glycerol and 0.1% propionate relied on tetracycline (ATc) induction of ICL expression. ICL KD without ATc was unable to grow because of the occlusion of the MCC caused by lacking ICL’s MCL activity. However, addition of at least 10 ng/mL ATc gradually induced ICL expression, with a subsequent release of sequestered carbons into the TCA cycle, restoring ICL KD growth and viability (Fig. S9A–C). Metabolomics profiles of ICL KD under varying doses of ATc showed a reciprocal relationship between the levels of MCC intermediates and E/Q values, inferring that WT Mtb relies on GltB/D mediated Q → E to buffer the negative metabolic consequences during hyperactive MCC (Fig. S9C). We extended the metabolomics findings of ICL KD to determine if co-inactivation of ICL’s MCL and GltB/D activities renders WT Mtb hypersensitive to G/P carbons. For this, we performed a dilution spot assay using ICL KD. At 1000 ng/mL ATc, ICL KD could form colonies regardless of carbon conditions but a lesser extent of colony formation was observed under the treatment of 1 µg/mL AZA only under G/P carbons. The level of colony formation was severely impaired following exposure to G/P carbons at 100 ng/mL or lower ATc and that was even exacerbated by additional treatment of AZA, compared to no AZA condition (Fig. 5D). We could similarly reproduce the synergistic bactericidal impact on ICL KD by CFU analysis (Fig. S9D). With intracellular ICL KD that were infected into THP-1, we also observed a hypersensitivity of intracellular ICL KD under the AZA treatment at the same level of ICL activity (Fig. 5E) without noticeable morphological changes or cytotoxicity in THP-1. These findings suggest that WT Mtb requires GltB/D mediated Q → E as a metabolic neutralization in order to efficiently assimilate propionate carbons through the MCC allowing Mtb to manage *in vitro* replication and adapt to host nutrient conditions. Furthermore, this crosstalk illuminates Mtb’s unique adaptive metabolism as a potential source of drug targets.

## Discussion

During the course of infection, Mtb is exposed to various nutrient conditions as phagocytosed Mtb bacillus inside alveolar macrophages are delivered deeper within the infected lung, ultimately forming granulomas^46^. Although it is advantageous that a wide range of nutrients are available to infected Mtb bacilli, this diversity is metabolically challenging^47^. Following phagocytosis of Mtb by macrophages, differential gene expression^48^ and metabolomics profiles^28^ have shown substantial upregulation of genes and their products involved in fatty acid degradation and metabolism *fad*, *gltA* (citrate synthase), *icl1*, *pckA* (PEP carboxykinase), *mez* (malate dehydrogenase); in MCC -*prpC*, *prpD*, *acn1*, and *icl1*; in cholesterol uptake and consumption *mce4* and *mce6*; and moderate upregulation of genes involved in gluconeogenesis including *ppdK* (pyruvate phosphate dikinase) and *fba* (fructose bisphosphate aldolase)^48^. Collectively, diverse species carbon sources ranging from cholesterol and fatty acids (acetate or propionate) to dextrose and glycerol are possible carbon sources for Mtb while residing inside hosts. *In vivo* experiments using Mtb harboring defined loss-of-function mutations in specific enzymes involved in carbon uptake and/or metabolism support this notion^26,29,49–53^. The success of Mtb as an intracellular human pathogen has thus been achieved by its ability to adapt to nutritionally changing environments.

Mtb’s metabolic plasticity for carbon uptake/consumption is now accepted as a hallmark of its virulence as a human pathogen. Recently appreciated multi-carbon co-catabolism is one example of the metabolic strategies used by Mtb to overcome the bioenergetic barrier during adaptation to host- implemented nutrient conditions^28^. The catabolic activities of cholesterol, fatty acids, and fermentable carbohydrates result in the unexpected accumulation of glyoxylate, propionyl-CoA, MCC intermediates and methylglyoxal with potentially toxic metabolic consequences^27,29,54,55^. Previous studies have shown the importance of the metabolic activities of pyruvate kinase (PYK) to neutralize unfavorable non-enzymatic metabolic side products, such as methylglyoxal that are produced during catabolizing ferment carbohydrate carbons^27^. Recently, Puckett *et al*. also revealed a new role of malate synthase (GlcB) in eliminating the accumulated glyoxylate and its mediated harmful consequences during assimilation of even-chain fatty acids through the glyoxylate shunt^55^. As such, Mtb is evolutionarily able to reprogram its metabolic networks toward efficient catabolism of different carbons in a thermodynamically favorable manner and for this, Mtb requires additional metabolic neutralization efforts to mitigate toxic consequences that arise during the assimilation processes within the context of interconnected metabolic networks.

Metabolic networks are subdivided into several highly linked and mathematically predictable functional units^56^. Delineation of these segments has been performed by genome-scale network reconstructions, but is somewhat biased by the scope and experimental conditions with limited integration of multiple hierarchical layers^57^. Interpretation of metabolic interactions helps reorganize the borders of these units that are shifted upon changing nutritional environments^58^. Sharing of the functional activities across regulatory structures can be triggered by metabolic networks that are reprogrammed depending upon available carbon sources, this metabolic plasticity is a potential driving force behind adaptive metabolism including emergence of drug-resistance. Evolutionary adaptive metabolism of Mtb permitting its survival in nutritionally adverse host environments may be achieved by these regulatory circuits. Conversely, BCG appears to have limited ability to adapt due to the lack of adaptive metabolism, potentially lost during manufacturing process. This may include the multiple genomic polymorphisms of BCG. Brosch *et al*. sequenced multiple BCG vaccine strains and identified that the phenotypic attenuation of BCG may partly be associated with multiple genetic insertion and/or deletion events including tandem duplications, DU1 and DU2, repetitive insertion elements, and SNPs in addition to deletion of RD1^16^. For example, nonsynonymous SNPs affect genes that are involved in mycolic acid biosynthesis (*mmaA3*)^59^, signaling pathway (*sigK*)^60^, central carbon metabolism (*pykA*)^61^ or transcriptional regulators (cAMP receptor protein)^62,63^. As such, BCG is metabolically distinct from Mtb in that it lacks the ability to catabolize glycerol and fatty acids (Fig. S1A); this defective metabolism can be associated with phenotypic attenuation. In this study, we showed that the phenotypic attenuation of BCG is mediated by failed adaptive metabolism, but is not associated with deficiency of RD1 locus. This finding is supported by previous studies, where RD1 deficient Mtb is attenuated compared to WT Mtb, but is still hypervirulent compared to BCG in a murine model^24,31,64^. This functional role of RD1 as a component for Mtb virulence is more for interaction with the host immune system rather than adaptation to host nutrients. Thus, our speculation was that since BCG is unable to conduct adaptive metabolism not because of a simple genetic deficiency, it serves an excellent model to define the mechanistic bases by which Mtb is able to manage its symbiont lifestyle.

Our study identified metabolomic profiles of WT Mtb during exposure to various carbon conditions (G/D or G/P) and provided an example of evolutionary metabolic networks that are unique to Mtb in catabolic utilization of gluconeogenic carbons such as propionate. We observed that Mtb maintains a lower pool size of toxic MCC intermediates during assimilation of propionate carbons by the ICL’s MCL catalytic activity, the last enzyme in the MCC. We also revealed that Mtb is equipped with GltB/D’s Q → E activity as a metabolic neutralization strategy to mitigate harmful consequences that occur while using MCC. As previously shown, with active Q → E, the conservation of glutamate pool should be expanded for following metabolic neutralizing activities that may include: **I** Gdh associated anaplerosis of the TCA cycle intermediates (glutamate to αKG) and **II** Gad mediated scavenging protons (glutamate to GABA)^38,65^. The impaired functional involvement of Q → E in BCG metabolism to dampen propionate toxicity is associated with its adaptive failure and rapid clearance from hosts. We proved this by the findings that the addition of VB12 and/or MSG was sufficient to protect BCG from the bactericidal effects due to MCC mediated propionate toxicity (Fig. 4). This is partly supported from a previous finding in that recombinant BCG expressing more copies of *icl1* had a higher survival rate in infected mice^66^. Recently, Mtb has shown to have an alternate route to mitigate the propionate toxicity; the biosynthesis of PDIM (phthiocerol dimycocerocates) through the drainage of toxic intermediates of the propionate metabolism^36^. Mtb may induce the PDIM biosynthesis as another source of metabolic effort to mitigate the propionate toxicity. However, our study showed that MSG supplement rescued BCG growth in media containing G/P to a level similar to WT Mtb growth, suggesting that crosstalk of MCC and GltB/D plays more crucial role in Mtb propionate metabolism^36^. Not only does maintenance of a glutamate pool play a role in metabolic neutralization including to buffer cytoplasmic pH, but also is known to be involved in the biosynthesis of γ-glutamylcysteine (Fig. S10A), a sulfur donor for a ubiquitous ergothioneine, which plays a crucial role in Mtb’s redox homeostasis. A recent report has confirmed that ergothioneine actively performs multiple regulatory functions including protecting against oxidative stress, balancing the levels of endogenous reactive oxygen species, and even conferring intrinsic antibiotic tolerance^67^. Furthermore, the content of ergothioneine in Mtb reflects the level of intrinsic redox stress of Mtb that is much greater when Mtb metabolizes cholesterol and a diverse set of fatty acid precursors^67^. Survival of intracellular Mtb is achieved by execution of adaptive metabolism, crosstalk between Q → E and MCC (Fig. S10B). The present work thus sheds light on one unique example of metabolic adaptation that occurs in Mtb during exposure to different host nutrients.

To date, the most attenuated Mtb mutants correspond to Mtb strains lacking annotated ICL^53^. ICL is a metabolic enzyme used by Mtb to sustain growth on fatty acids through two pathways: the glyoxylate shunt and MCC^29,30^. Recent studies have shown that ICL is also essential for Mtb to both establish and maintain infection, when it is thought to persist in a metabolically quiescent state^68,69^ and resist to multiple bactericidal anti-TB drugs^70,71^. Thus, it is generally accepted that Mtb ICL is a potential drug target to kill Mtb in diverse metabolic states. Efforts to identify pharmacologically relevant ICL inhibitors, however, have been unsuccessful because of fundamental gaps in our knowledge about the impacts of ICL deficiency on Mtb metabolism. We recently provided a systems level insight into the essentiality of Mtb ICL and revealed a functional link between MCC-mediated propionate toxicity and associated impaired membrane bioenergetics^29^. The results of this study additionally provide evidence of the GltB/D’s Q → E mediated metabolic neutralization efforts to dampen ICL’s MCL deficiency associated propionate toxicity on Mtb metabolism. Collectively, we suggest that previously screened, but pharmacologically irrelevant ICL inhibitors, need to be retested under a new experimental setting in which Q → E activity is interfered.

In summary, metabolic adaptation in response to changing environments is a physiological prerogative of all biological systems and requires functional plasticity and crosstalk of metabolic enzymes. This study reveals the evidence that Mtb uses metabolic adaptation strategies to survive intracellular nutrient environments. The interactive crosstalk between MCC and the Q-E pathway can’t be deduced by genetic information, thus exemplifying the importance of metabolomics to resolve the essential metabolic capabilities of intracellular WT Mtb. From a pharmacological perspective, inhibition of this metabolic crosstalk by combinatorial inhibition in WT Mtb will be effective in eradicating WT Mtb by preventing efficient adaptation to the host environments (Fig. S10B).

## Methods

### Bacterial strains and culture conditions

*Mycobacterium bovis* bacillus Calmette-Guérin (BCG) Pasteur (1173P2), Swedish (TMC 1009), Danish (TMC 1010), Mtb H_37_Rv (WT Mtb), and *icl* knock-down (ICL KD) Mtb strain were cultured in either Middlebrook (m) 7H9 broth or m7H10 agar media (Difco, Detroit, MI) supplemented with 0.5% fraction V bovine serum albumin (BSA), 0.085% NaCl, 0.04% tyloxapol (broth only) with 0.2% glycerol, dextrose or varying concentrations (0–0.8% vol/vol) of propionate. For culturing *M*. *bovis* and ΔRD1, both m7H9 broth or m7H10 agar media was generated with aforementioned supplements and additional sodium pyruvate. For generation of acidic agar plates (pH 5.5), m7H9 broth was solidified by adding 15% w/v Bactoagar after pH adjustment using HCl. For metabolomic profiling, WT Mtb, BCG, *M*. *bovis*, or ΔRD1-laden filters were generated as previously described^28,68^. Mtb strains of H_37_Rv, ICL KD, *M*. *bovis* and ΔRD1 were cultured in a containment of biosafety level 3 facility.

### Metabolite extraction and chemicals

WT Mtb, ICL KD, BCG, *M*. *bovis*, or ΔRD1-laden filters were incubated at 37 °C for 5 days to reach the midlogarithmic phase of growth. The filters were then transferred onto chemically identical m7H10 agar containing fresh carbon sources and metabolites were harvested after 24 hrs. WT Mtb, BCG, *M*. *bovis*, or ΔRD1 was metabolically quenched by plunging the bacteria-laden filters into LC-MS-grade acetonitrile:methanol:water (40:40:20) solution that was precooled to −40 °C. Metabolites were extracted by mechanical lysis with 0.1 mm Zirconia beads in a Precellys tissue homogenizer (Bertin Technologies, France) for 8 min (6,800 rpm) twice under continuous cooling at or below 2 °C. Lysates were clarified by centrifugation and then filtered across 0.22 µm Spin-X columns. Residual protein content of metabolite extracts was measured (BCA protein assay kit; Thermo Scientific, Waltham, MA, USA) to normalize samples to cell biomass. Itaconic acid, azaserine, vitamin B12, monosodium glutamate, and sodium pyruvate were purchased from Sigma-Aldrich (Saint Louis, MO, USA) and used at 2 mM, 1.0 µg/mL, 10 µg/mL, 10 mM, and 40 mM respectively.

### Metabolomics with LC-MS

Liquid chromatography mass spectrometry (LC-MS) differentiation and detection of WT Mtb, ICL knock-down, BCG, *M*. *bovis*, and ΔRD1 metabolites were performed with an Agilent Accurate Mass 6230 TOF coupled with an Agilent 1290 Liquid Chromatography system using a Cogent Diamond Hydride Type C column (Microsolve Technologies, Long Branch, NJ, USA) using solvents and configuration as previously described^68^. An isocratic pump was used for continuous infusion of a reference mass solution to allow mass axis calibration. Detected ions were classified as metabolites based on unique accurate mass-retention time identifiers for masses showing the expected distribution of accompanying isotopologues. Metabolites were analyzed using Agilent Qualitative Analysis B.07.00 and Profinder B.06.00 software (Agilent Technologies, Santa Clara, CA, USA) with a mass tolerance of <0.005 Da. Standards of authentic chemicals of known amounts were mixed with bacterial lysates and analyzed to generate the standard curves used to quantify metabolite levels. All data obtained by metabolomics were the average of at least two independent triplicates for each condition tested.

### Human macrophage infection and metabolomics

THP-1 (human monocyte cell line) were cultured in RPMI1640 supplemented with 10% FBS and differentiated to macrophages by treatment of 100 ng/mL of TPA (12-O-tetra-decanoylphorbol-13-acetate) for 48 hrs at 37 °C and 5% CO_2_. Undifferentiated cells were removed by washing with prewarmed PBS three times. For infection, early logarithmic phase bacterial cultures were prepared by dilution to a predefined viable number of bacilli in antibiotic-free RPMI. THP-1 then were infected at a multiplicity of infection (MOI) of 1.0 of CFU-precalibrated BCG and WT Mtb. Uninfected bacilli were removed by PBS after 4 hrs. Infected cells were washed three times with prewarmed PBS and extracellular bacilli were killed by overnight treatment with 30 µg/mL gentamycin. Cells were lysed with 0.5% Triton X-100 and released bacilli were enumerated by plating serial dilutions of the lysate on m7H10 agar plates 0 and 5 days post-infection.

For extraction of metabolites, 1 × 10^6^ THP-1 were seeded for infection with either BCG or WT Mtb at MOI 5.0, and for treatment with 10 µg/mL LPS (Sigma-Aldrich) in triplicate in 6 well plates. After a 24-hour infection, supernatants were removed and adherent cells were thoroughly washed with ice-cold PBS. Released bacilli were removed by centrifugation and filtration through 0.2 µm Spin-X columns. Cells were then lysed with 1 mL of cold 80% methanol with or without pretreatment with 0.025% SDS (to remove infected bacilli). Either total metabolome or bacilli free metabolome was extracted at −80 °C overnight and subsequently analyzed by LC-MS^33^.

### RNA extraction for qrtPCR

WT Mtb or BCG were grown on filter membranes as done in metabolite extraction, and exposed to various carbon conditions (G/D or G/P carbons) for 24 hours. Total RNA was extracted by scraping the bacilli into TRIZOL solution and mechanically lysing with 0.1 mm Zirconia beads in a Precellys tissue homogenizer. Lysates were clarified by centrifugation and TRIZOL supernatant was removed and used for RNA extraction. RNA was isolated using a Qiagen RNA extraction kit. Isolated RNA was treated with DNase I to remove DNA contamination (Sigma). RNA concentrations were determined using a Nanodrop and qrtPCR reactions were conducted using a iQ SYBR-Green Supermix (BioRad, Hercules, CA, USA) and analyzed by C1000^TM^ Thermal Cycler Instrument. The primers used for amplification were 5′-GCACTGTGGCACAAGATTTC-3′ (forward) and 5′-TTCGTCCACGATCACTTCAC-3′ (reverse) for *prp*D, 5′-GCGTACTACCTGATGGGATTC-3′ (forward) and 5′-CGTGGCCTGTTCCATGAT-3′ (reverse) for *prp*C, and 5′-ATCGCCAAGTTCCAGAAGG-3′ (forward) and 5′-GCCAGATCGAACATCGAGTAG-3′ (reverse) for *icl*1. Fold changes were calculated by Δ*C*_*T*_ values that were normalized to *sigA* transcript levels and depicted as log_2_ values relative to growth permissive carbon condition as controls.

### *In vitro* glutamate synthase (GltB/D) assay

Cytoplasmic fractions were prepared from Mtb or BCG laden filters (cultured similarly to samples used for metabolomics profiling). Mtb or BCG bacilli were resuspended in 1 mL Tris-Cl (pH 7.4) and transferred to 2 mL screw-cap tubes containing 0.4 mL of 0.1 mm diameter Zirconia beads. Cells were disrupted by 30s pulses in Precellys tissue homogenizer for 30s pulses three times (6,000 rpm) twice under continuous cooling at or below 2 °C. Lysates were then centrifuged at 13,000 g for 10 min at 4 °C. and supernatants were subsequently transferred to fresh tubes. Concentration of cytoplasmic fractions were determined by BCA protein assay kit (Thermo Scientific, Waltham, MA, USA). *In vitro* GltB/D reactions were performed with a 30 µL reaction volumes containing 100 mM glycine-KOH (pH 8.5), 2 mM glutamine, 2 mM αKG, 5 mM MgSO_4_, and 0.25 mM NADPH. The reactions were initiated by adding 0.1 µg of cell lysates and quenched by adding 70 µL of 100% acetonitrile with 0.2% formic acid. The production of glutamate was monitored by LC-MS. The 2MI mediated GltB/D inhibition was measured by adding various concentrations of 2MI (0.2~2.0 mM) in each reaction prior to addition of cell lysate.

### CFU assay and bacterial growth chase

Midlogarithmic phase of Mtb or BCG was used. An OD_595_ 0.05 Mtb or BCG was incubated in m7H9 broth containing various carbon sources in 24-well microplates. Bacterial cultures were then serially diluted and plated on m7H10 agar with supplements: 0.5% glycerol, 0.2% dextrose, 0.5 g/L BSA, and 0.085% w/v NaCl for 3 weeks at 37 °C. Growth was monitored by measuring the OD_595_ of the liquid cultures by using GENESYS^TM^ 20 (Thermo Scientific). All assays were performed as two independents with triplicates.

### Determination of intrabacterial pH

We used Mtb expressing a reported, pH-sensitive ratiometric green fluorescent protein (BCG Pasteur-pHGFP) as previously described^72^. BCG-pHGFP cultures were grown to midlogarithmic phase (OD_595_ ~ 0.6) and resuspended in m7H9 with 0.2% glycerol and various concentrations of propionate (0 to 0.8%) either with or without 2 mM itaconic acid and 1 µg/mL azaserine at various time points. Fluorescence was measured using a FLUOstar Omega plate reader (BGM LABTECH Inc., Cary, NC, USA) at excitation 355 nm, emission 520 nm (reading 1) and at excitation 485 nm, emission 520 nm (reading 2). Intrabacterial pH was inferred from the ratio of reading 1 to reading 2 by reference to a calibration curve performed on lysates of BCG-pHGFP as described^72^.

### Preparation of purified recombinant ICL1

The *icl1* gene (*rv0467*) encoding ICL1 was amplified by PCR using the primer set; forward, 5′-CAT ATG TCT GTC GTC GGC ACC CCG A-3′ and reverse, 5′-AAG CTT CTA GTG GAA CTG GCC CTC TTC GGT GGA-3′ (*Nde*I and *Hin*dIII restriction site underlined). The amplified gene and pET28a (Novagen, Madison, WI, USA) were double digested with *Nde*I and *Hin*dIII and ligated to construct pET28a::*icl1*. Once the sequence was confirmed, *E*. *coli* BL21(DE3) was used as an expression host. A hexa-His ICL1 was purified by standard Ni-NTA chromatography as performed elsewhere. Protein was dialyzed, concentrated, aliquoted, and stored at −80 °C. The final buffer contained 20 mM Tris-Cl (pH 7.4), 1 mM DTT, 175 mM NaCl and the purity was confirmed by a Coomassie Blue staining assay.

### Activity based metabolomics profiling

Small metabolite extracts of Mtb and BCG were prepared as previously described^73^. Mtb or BCG was grown in 100 mL m7H9 with ADN. The pellet was suspended in 1 mL of acidic ACN solution (acetonitrile, 0.2% formic acid) and cells were disrupted by bead beating. The soluble fraction was obtained by centrifugation at 20,000 g for 10 min at 4 °C, flash-freezing, and lyophilizing. Half of the dry SME was resuspended in 1 mL of 20 mM Tris-Cl (pH 7.4) and the insoluble fraction removed by centrifugation. Aliquots were stored at – 80 °C. Samples (typically 10 µL) of the SME were incubated for various times with or without 600 nM recombinant purified ICL1, and 1 mM Mg^2+^ in a final volume of 100 uL of 20 mM Tris-Cl. Cold ACN containing 0.2% formic acid was added for quenching, yielding 70% ACN final mixtures. After centrifugation at 20,000 g for 10 min at 4 °C, samples were kept at −80 °C until analyzed by LC-MS as described earlier.

### Generation of ΔRD1 mutant

Recombineering technology was employed to delete the RD1 locus (ΔRD1) in the chromosome of *M*. *bovis*, as was previously used to delete RD1 in Mtb^31^. Briefly, genomic regions (~800 bp each) flanking the RD1 locus were amplified and cloned into the pKO (pKO-RD1) to replace RD1 with the Kan^R^ cassette and pKO-RD1 was introduced into *M*. *bovis* by electroporation. Colonies were selected on m7H10 plates containing 10% sucrose and hygromycin (100 µg/mL). Once confirmed as single cross-over candidates, double cross-over candidates were selected by checking the selective sensitivity against hygromycin but resistance to both 10% sucrose and kanamycin (50 µg/mL). Final colonies were tested by both whole genome sequencing (ChunLab, Inc., Seoul National University, South Korea) and multiplex PCR^32^ as used to screen for RD1 depletion in many *Mycobacterium spp*. including *M*. *bovis* and BCG.

### Dilution spot assay

After ICL knock-down at midlogarithmic phase was diluted to OD_595_ 0.5, the culture was further diluted 10-fold and 2 µL was spotted onto m7H9 medium solidified by agar containing either G/D or G/P carbons in the presence varying doses of ATc (0, 10, 100, and 1000 ng/mL). 0 or 1 µg/mL azaserine was additionally added and the plates were incubated at 37 °C until colony formation.

### Chemical synthesis of D,L 2-methylisocitrate

NMR spectra were recorded on a Bruker Advance 500 (^1^H 500 MHz, ^13^C 125 MHz) or 400 (^1^H 400 MHz, ^13^C 100 MHz) spectrometer. Chemical shifts (δ) in parts per million are given relative to Me_4_Si, coupling constants (J) are given in hertz. Analytical TLC was carried out in either Merck Silica 60 F254 or RP-18 F254s plates. The components were observed under ultraviolet light (254 nm) and/or dipped in 5% H_2_SO_4_ in MeOH followed by heating using a heat gun. Other chemicals used were purchased from Sigma-Aldrich or Alfa Aesar (Lancashire, UK) unless otherwise stated. Synthesis of D,L-theo-2-methyl isocitrate was as described by Munoz-Elias *et al*.^74^ with a few modifications.

*Trans*-*epoxymethylsuccinic acid* (**2**)*:* Mesaconate (**1**) (1.0 g, 7.88 mmol) was suspended in water (5 mL) and NaOH (0.61 g, 15.37 mmol) was added dropwise with stirring. The mixture was stirred and cooled to 0 °C and Na_2_WO_4_-2H_2_O (76 mg, 0.23 mmol) was added followed by dropwise addition of a 30% aqueous solution of H_2_O_2_ (1 mL, 8.50 mmol). After stirring at 0 °C for 1 hr, the mixture was heated at 65 °C for 8 hrs, then cooled to RT and evaporated to dryness. The residue was partitioned between water (10 mL), ethyl acetate (20 mL) and ether (20 mL). Concentrated H_2_SO_4_ (1 mL) dissolved in ether (10 mL) was added and the mixture stirred at RT for 3hrs. The organic layer was separated, dried (Na_2_SO_4_) and evaporated to afford the epoxide **2** as a white powder in quantitative yield. ^1^H NMR (400 MHz, DMSO-*d*_6_) δ 13.44 (s, 2 H), 3.75 (s, 1 H), 1.45 (s, 3 H).

*Dimethyl trans*-*epoxymethylsuccinate* (**3**). To a solution of epoxide (**2**) (1.0 g, 6.84 mmol) in anhydrous MeOH (10 mL) at 0 °C, was added SO_2_CI_2_ (1.1 mL, 15.0 mmol) and the resulting mixture allowed to warm up to room temperature and stirred for 8 hrs. The reaction mixture was concentrated under vacuum and the residue partitioned between ether and saturated aqueous NaHCO_3_. The ether layer was separated, dried and evaporated to afford the dimethyl ester **3** as a clear oil in quantitative yield and used without any further purification. ^1^H NMR (500 MHz, DMSO-*d*_6_) δ 6.46 (d, *J* = 7.2 Hz, 1H), 6.09 (s, 1H), 4.94 (s, 1H), 4.62 (d, *J* = 7.2 Hz, 1H), 3.74 (s, 3H), 3.69 (s, 2H), 3.67 (d, *J* = 4.2 Hz, 5H), 1.71 (s, 3H), 1.44 (s, 3H).

*2*-*Methyl*-*5*-*oxo*-*tetrahydro*-*2*,*3*,*4*-*tricarbomethoxytetrahydrofuran* (**4**). Sodium metal (0.69 g, 28.7 mmol) was added to cooled anhydrous MeOH (15 mL) under argon atmosphere at 0 °C and stirred until the metal dissolved. Dimethyl malonate (3.2 mL, 28.7 mmol) was added to the sodium methoxide thus formed, and the resulting mixture was stirred at RT until a white precipitate formed (20–30 min). Epoxide **3** (5.0 g, 28.7 mmol) was added and the mixture stirred for 3 days at RT. Concentrated HCI was added and the stirring continued for another 2 hrs. The solid formed (NaCI) was filtered and the filtrate concentrated to afford crude lactone **4** as a white solid in good yield and used without any further purification.

*DL*-*threo*-*2*-*methylisocitrate* (**6**). To the crude lactone **4** obtained above (1.5 g) 6 N HCI (50 mL) was added and the mixture refluxed at RT for 3 hrs. The reaction mixture was concentrated; to remove traces of HCI, the residue was redissolved in water and evaporated. The brown residue thus obtained was dissolved in water and treated with activated charcoal at 60 °C. After 30 min, the mixture was filtered and the filtrate evaporated to afford colorless crude lactone *2*-*Methyl*-*5*-*oxo*-*tetrahydrofuran*-*2*,*3*-*dicarboxylic acid* (**5**). To a solution of lactone **5** (1.5 g, 7.98 mmol) in H_2_O (10 mL), NaOH (0.95 g, 23.9 mmol) was added and the mixture heated at 80 °C for 12 hrs, then neutralized with Amberlite H^+^ resin to pH 7.0. After solvent removal, the residue was redissolved in H_2_O and treated with activated charcoal. The mixture was filtered and the filtrated evaporated to afford D,L-*threo*-2-methylisocitrate **6** in a good yield (60%).

## Electronic supplementary material

Fig. S1-S10, Table 1, 2

## References

[1] WHO. Global tuberculosis report 2017 (2017).

[2] Gandhi NR (2010). Multidrug-resistant and extensively drug-resistant tuberculosis: a threat to global control of tuberculosis. Lancet.

[3] Velayati AA (2009). Emergence of new forms of totally drug-resistant tuberculosis bacilli: super extensively drug-resistant tuberculosis or totally drug-resistant strains in iran. Chest.

[4] Smith I (2003). Mycobacterium tuberculosis pathogenesis and molecular determinants of virulence. Clin Microbiol Rev.

[5] Olive AJ, Sassetti CM (2016). Metabolic crosstalk between host and pathogen: sensing, adapting and competing. Nat Rev Microbiol.

[6] Abu Kwaik Y, Bumann D (2013). Microbial quest for food *in vivo*: ‘nutritional virulence’ as an emerging paradigm. Cell Microbiol.

[7] Zhang YJ, Rubin EJ (2013). Feast or famine: the host-pathogen battle over amino acids. Cell Microbiol.

[8] Fonseca MV, Swanson MS (2014). Nutrient salvaging and metabolism by the intracellular pathogen *Legionella pneumophila*. Front Cell Infect Microbiol.

[9] Hood MI, Skaar EP (2012). Nutritional immunity: transition metals at the pathogen-host interface. Nat Rev Microbiol.

[10] Gianoulis TA (2009). Quantifying environmental adaptation of metabolic pathways in metagenomics. Proc Natl Acad Sci USA.

[11] Narayanaswamy R (2009). Widespread reorganization of metabolic enzymes into reversible assemblies upon nutrient starvation. Proc Natl Acad Sci USA.

[12] Grange JM (2000). Effective vaccination against tuberculosis-a new ray of hope. Clin Exp Immunol.

[13] Layre E (2011). A comparative lipidomics platform for chemotaxonomic analysis of *Mycobacterium tuberculosis*. Chem Biol.

[14] Layre E (2014). Molecular profiling of *Mycobacterium tuberculosis* identifies tuberculosinyl nucleoside products of the virulence-associated enzyme Rv3378c. Proc Natl Acad Sci USA.

[15] Garnier T (2003). The complete genome sequence of *Mycobacterium bovis*. Proc Natl Acad Sci USA.

[16] Brosch R (2007). Genome plasticity of BCG and impact on vaccine efficacy. Proc Natl Acad Sci USA.

[17] Brosch R (2002). A new evolutionary scenario for the *Mycobacterium tuberculosis* complex. Proc Natl Acad Sci USA.

[18] Mostowy S, Cousins D, Brinkman J, Aranaz A, Behr MA (2002). Genomic deletions suggest a phylogeny for the *Mycobacterium tuberculosis* complex. J Infect Dis.

[19] Sorensen AL, Nagai S, Houen G, Andersen P, Andersen AB (1995). Purification and characterization of a low-molecular-mass T-cell antigen secreted by *Mycobacterium tuberculosis*. Infect Immun.

[20] Berthet FX, Rasmussen PB, Rosenkrands I, Andersen P, Gicquel BA (1998). *Mycobacterium tuberculosis* operon encoding ESAT-6 and a novel low-molecular-mass culture filtrate protein (CFP-10). Microbiology.

[21] Cole ST (1998). Deciphering the biology of *Mycobacterium tuberculosis* from the complete genome sequence. Nature.

[22] Hsu T (2003). The primary mechanism of attenuation of bacillus Calmette-Guerin is a loss of secreted lytic function required for invasion of lung interstitial tissue. Proc Natl Acad Sci USA.

[23] Stanley SA, Raghavan S, Hwang WW, Cox JS (2003). Acute infection and macrophage subversion by *Mycobacterium tuberculosis* require a specialized secretion system. Proc Natl Acad Sci USA.

[24] Guinn KM (2004). Individual RD1-region genes are required for export of ESAT-6/CFP-10 and for virulence of *Mycobacterium tuberculosis*. Mol Microbiol.

[25] Behr MA (2002). BCG–different strains, different vaccines?. Lancet Infect Dis.

[26] Puckett S (2014). Inactivation of fructose-1,6-bisphosphate aldolase prevents optimal co-catabolism of glycolytic and gluconeogenic carbon substrates in *Mycobacterium tuberculosis*. PLoS Pathog.

[27] Noy T (2016). Central Role of Pyruvate Kinase in Carbon Co-catabolism of *Mycobacterium tuberculosis*. J Biol Chem.

[28] de Carvalho LP (2010). Metabolomics of *Mycobacterium tuberculosis* reveals compartmentalized co-catabolism of carbon substrates. Chem Biol.

[29] Eoh H, Rhee KY (2014). Methylcitrate cycle defines the bactericidal essentiality of isocitrate lyase for survival of *Mycobacterium tuberculosis* on fatty acids. Proc Natl Acad Sci USA.

[30] Gould TA, van de Langemheen H, Munoz-Elias EJ, McKinney JD, Sacchettini JC (2006). Dual role of isocitrate lyase 1 in the glyoxylate and methylcitrate cycles in *Mycobacterium tuberculosis*. Mol Microbiol.

[31] Lewis KN (2003). Deletion of RD1 from *Mycobacterium tuberculosis* mimics bacille Calmette-Guerin attenuation. J Infect Dis.

[32] Kim Y (2013). A simple and efficient multiplex PCR assay for the identification of Mycobacterium genus and *Mycobacterium tuberculosis* complex to the species level. Yonsei Med J.

[33] Dey B (2015). A bacterial cyclic dinucleotide activates the cytosolic surveillance pathway and mediates innate resistance to tuberculosis. Nat Med.

[34] de Carvalho LP (2010). Activity-based metabolomic profiling of enzymatic function: identification of Rv1248c as a mycobacterial 2-hydroxy-3-oxoadipate synthase. Chem Biol.

[35] de Carvalho LP, Darby CM, Rhee KY, Nathan C (2011). Nitazoxanide Disrupts Membrane Potential and Intrabacterial pH Homeostasis of *Mycobacterium tuberculosis*. ACS Med Chem Lett.

[36] Lee W, VanderVen BC, Fahey RJ, Russell DG (2013). Intracellular *Mycobacterium tuberculosis* exploits host-derived fatty acids to limit metabolic stress. J Biol Chem.

[37] Savvi S (2008). Functional characterization of a vitamin B12-dependent methylmalonyl pathway in *Mycobacterium tuberculosis*: implications for propionate metabolism during growth on fatty acids. J Bacteriol.

[38] Gallant JL, Viljoen AJ, van Helden PD, Wiid IJ (2016). Glutamate Dehydrogenase Is Required by *Mycobacterium bovis* BCG for Resistance to Cellular Stress. PLoS One.

[39] Choi JW (2015). Enhanced production of gamma-aminobutyrate (GABA) in recombinant *Corynebacterium glutamicum* by expressing glutamate decarboxylase active in expanded pH range. Microb Cell Fact.

[40] Karatzas KA, Brennan O, Heavin S, Morrissey J, O’Byrne CP (2010). Intracellular accumulation of high levels of gamma-aminobutyrate by *Listeria monocytogenes* 10403S in response to low pH: uncoupling of gamma-aminobutyrate synthesis from efflux in a chemically defined medium. Appl Environ Microbiol.

[41] Cotter PD, Gahan CG, Hill C (2001). A glutamate decarboxylase system protects *Listeria monocytogenes* in gastric fluid. Mol Microbiol.

[42] Richard H, Foster JW (2004). *Escherichia coli* glutamate- and arginine-dependent acid resistance systems increase internal pH and reverse transmembrane potential. J Bacteriol.

[43] Bearson BL, Lee IS, Casey TA (2009). *Escherichia coli* O157: H7 glutamate- and arginine-dependent acid-resistance systems protect against oxidative stress during extreme acid challenge. Microbiology.

[44] Lamichhane G (2011). Essential metabolites of *Mycobacterium tuberculosis* and their mimics. MBio.

[45] Blumenthal A, Trujillo C, Ehrt S, Schnappinger D (2010). Simultaneous analysis of multiple *Mycobacterium tuberculosis* knockdown mutants *in vitro* and *in vivo*. PLoS One.

[46] Russell DG (2010). *Mycobacterium tuberculosis* wears what it eats. Cell Host Microbe.

[47] Eisenreich W, Dandekar T, Heesemann J, Goebel W (2010). Carbon metabolism of intracellular bacterial pathogens and possible links to virulence. Nat Rev Microbiol.

[48] Schnappinger D (2003). Transcriptional Adaptation of *Mycobacterium tuberculosis* within Macrophages: Insights into the Phagosomal Environment. J Exp Med.

[49] Ganapathy U (2015). Two enzymes with redundant fructose bisphosphatase activity sustain gluconeogenesis and virulence in *Mycobacterium tuberculosis*. Nat Commun.

[50] Marrero J, Rhee KY, Schnappinger D, Pethe K, Ehrt S (2010). Gluconeogenic carbon flow of tricarboxylic acid cycle intermediates is critical for *Mycobacterium tuberculosis* to establish and maintain infection. Proc Natl Acad Sci USA.

[51] Marrero J, Trujillo C, Rhee KY, Ehrt S (2013). Glucose phosphorylation is required for *Mycobacterium tuberculosis* persistence in mice. PLoS Pathog.

[52] Rhee KY (2011). Central carbon metabolism in *Mycobacterium tuberculosis*: an unexpected frontier. Trends Microbiol.

[53] Munoz-Elias EJ, McKinney JD (2005). *Mycobacterium tuberculosis* isocitrate lyases 1 and 2 are jointly required for *in vivo* growth and virulence. Nat Med.

[54] Maksymiuk C, Balakrishnan A, Bryk R, Rhee KY, Nathan CF (2015). E1 of alpha-ketoglutarate dehydrogenase defends *Mycobacterium tuberculosis* against glutamate anaplerosis and nitroxidative stress. Proc Natl Acad Sci USA.

[55] Puckett S (2017). Glyoxylate detoxification is an essential function of malate synthase required for carbon assimilation in *Mycobacterium tuberculosis*. Proc Natl Acad Sci USA.

[56] Stelling J, Klamt S, Bettenbrock K, Schuster S, Gilles ED (2002). Metabolic network structure determines key aspects of functionality and regulation. Nature.

[57] Monk J, Nogales J, Palsson BO (2014). Optimizing genome-scale network reconstructions. Nat Biotechnol.

[58] Rhee K (2013). Minding the gaps: metabolomics mends functional genomics. EMBO Rep.

[59] Belley A (2004). Impact of methoxymycolic acid production by *Mycobacterium bovis* BCG vaccines. Infect Immun.

[60] Charlet D (2005). Reduced expression of antigenic proteins MPB70 and MPB83 in *Mycobacterium bovis* BCG strains due to a start codon mutation in *sigK*. Mol Microbiol.

[61] Keating LA (2005). The pyruvate requirement of some members of the *Mycobacterium tuberculosis* complex is due to an inactive pyruvate kinase: implications for *in vivo* growth. Mol Microbiol.

[62] Hunt DM (2008). Single nucleotide polymorphisms that cause structural changes in the cyclic AMP receptor protein transcriptional regulator of the tuberculosis vaccine strain *Mycobacterium bovis* BCG alter global gene expression without attenuating growth. Infect Immun.

[63] Garcia Pelayo MC (2009). A comprehensive survey of single nucleotide polymorphisms (SNPs) across *Mycobacterium bovis* strains and *M*. *bovis* BCG vaccine strains refines the genealogy and defines a minimal set of SNPs that separate virulent *M*. *bovis* strains and *M*. *bovis* BCG strains. Infect Immun.

[64] Sherman DR (2004). *Mycobacterium tuberculosis* H37Rv: Delta RD1 is more virulent than *M*. *bovis* bacille Calmette-Guerin in long-term murine infection. J Infect Dis.

[65] Feehily C, Karatzas KA (2013). Role of glutamate metabolism in bacterial responses towards acid and other stresses. J Appl Microbiol.

[66] Szabo AM, Endresz V, Somogyvari F, Miczak A, Faludi I (2013). Isocitrate lyase encoding plasmids in BCG cause increased survival in ApoB100-only LDLR−/− mice. Mol Biol Rep.

[67] Saini V (2016). Ergothioneine Maintains Redox and Bioenergetic Homeostasis Essential for Drug Susceptibility and Virulence of *Mycobacterium tuberculosis*. Cell Rep.

[68] Eoh H, Rhee KY (2013). Multifunctional essentiality of succinate metabolism in adaptation to hypoxia in *Mycobacterium tuberculosis*. Proc Natl Acad Sci USA.

[69] Gengenbacher M, Rao SP, Pethe K, Dick T (2010). Nutrient-starved, non-replicating *Mycobacterium tuberculosis* requires respiration, ATP synthase and isocitrate lyase for maintenance of ATP homeostasis and viability. Microbiology.

[70] Nandakumar M, Nathan C, Rhee KY (2014). Isocitrate lyase mediates broad antibiotic tolerance in *Mycobacterium tuberculosis*. Nat Commun.

[71] Kohanski MA, Dwyer DJ, Hayete B, Lawrence CA, Collins JJ (2007). A common mechanism of cellular death induced by bactericidal antibiotics. Cell.

[72] Vandal OH, Pierini LM, Schnappinger D, Nathan CF, Ehrt S (2008). A membrane protein preserves intrabacterial pH in intraphagosomal *Mycobacterium tuberculosis*. Nat Med.

[73] Nandakumar M, Prosser GA, de Carvalho LP, Rhee K (2015). Metabolomics of *Mycobacterium tuberculosis*. Methods Mol Biol.

[74] Munoz-Elias EJ, Upton AM, Cherian J, McKinney JD (2006). Role of the methylcitrate cycle in *Mycobacterium tuberculosis* metabolism, intracellular growth, and virulence. Mol Microbiol.

