# Supplementary material for: Glutamate mediated metabolic neutralization mitigates propionate toxicity in intracellular Mycobacterium tuberculosis
Source: Sci Rep. 2018 May 31;8:8506. doi: 10.1038/s41598-018-26950-z (PMC5981324; doi:10.1038/s41598-018-26950-z)
Supplement: Supplementary file 1 — Fig. S1-S10, Table 1, 2 [file 41598_2018_26950_MOESM1_ESM.pdf]

**Extended Data for Lee et al.**

# Glutamate mediated metabolic neutralization mitigates propionate toxicity in intracellular *Mycobacterium tuberculosis*.

Jae Jin Lee, Juhyeon Lim, Shengjia Gao, Christopher P. Lawson, Mark Odell, Saki Raheem, JeongIm Woo, Sung-Ho Kang, Shin-Seok Kang, Bo-Young Jeon, Hyungjin Eoh

This document contains Supplement Figure Legends S1 - S10 and Table Legends S1 – S2.

Supplementary figures and legends

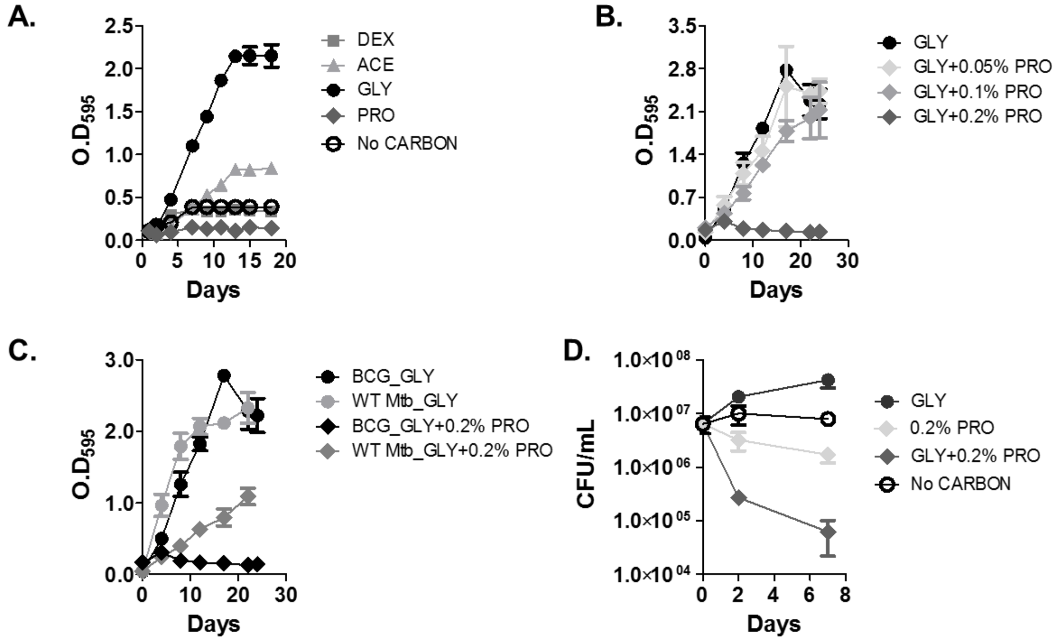


**Fig. S1.**

**Carbon source dependent phenotypic characterization of WT Mtb ($H_{37}$Rv) and BCG (Pasteur 1173P2)**. Growth phenotype of BCG (reported by $O.D_{595}$) cultured in media containing various single carbon sources (at 0.2% concentration) (**A**) and 0.2% glycerol supplemented with varying concentrations of propionate (0.05%, 0.1% and 0.2%) (**B**). (**C**) Growth phenotypes of WT Mtb and BCG cultured in media containing 0.2% glycerol and the effect of adding 0.2% propionate. (**D**) Effect of propionate carbon source on CFU-based viability of BCG cultured in m7H9 containing 0.2% of the indicated carbon sources. All values are the average of two independent experiments, each of which consisted of biological triplicates, ±SD (**A**, **B**, and **C**) and ±s.e.m (**D**).

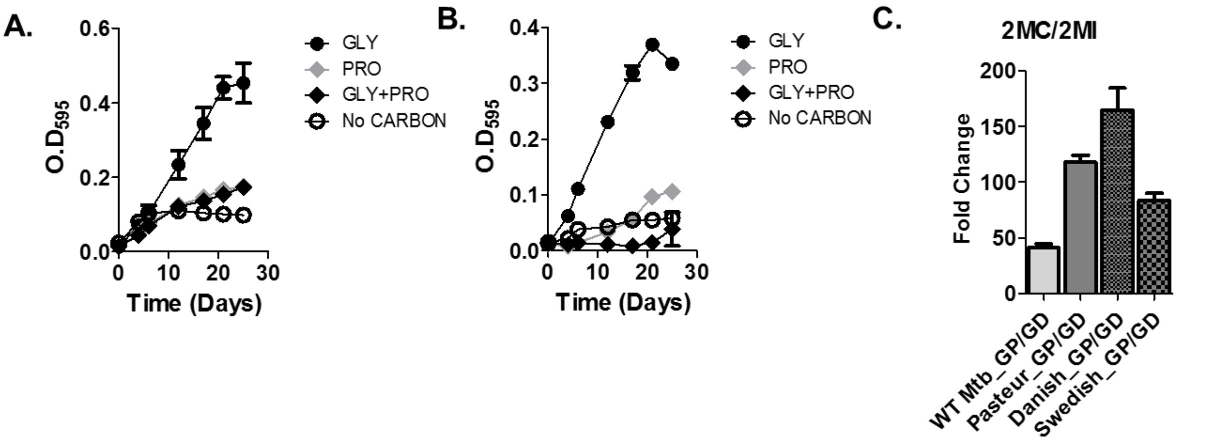


**Fig. S2.**

Growth phenotypes of BCG Danish strain (**A**) and BCG Swedish strain (**B**) in media containing various carbon sources (GLY, 0.2% glycerol; PRO, 0.2% propionate; GLY+PRO, 0.2% glycerol + 0.2% propionate). All values are the average of two independent experiments, each of which consisted of biological triplicates, ±SD.

(**C**) Metabolomics profiles of 2MC/2MI (2-methylcitrate/2-methylisocitrate) of BCG Pasteur, Danish, and Swedish strains relative to 2MC/2MI of WT Mtb under G/P carbons. All values are the average of two independent experiments, each of which consisted of biological triplicates, ±s.e.m.

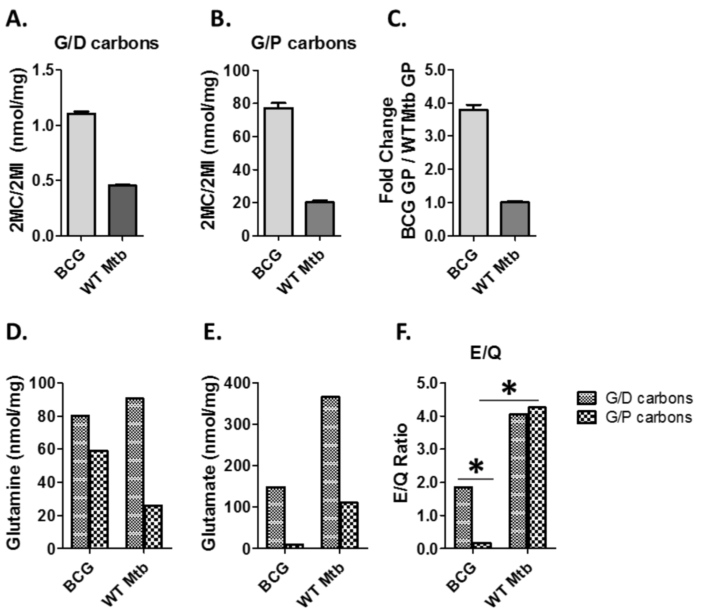


**G**. Summary of E, Q, and E/Q

| µg/mL | | WT Mtb | | BCG | |
|---|---|---|---|---|---|
| | | E | Q | E | Q |
| G/D | | 366.7 | 90.6 | 148.7 | 80.1 |
| G/P | | 111.3 | 26.1 | 9.9 | 58.9 |
| E/Q | G/D | 4.04 | | 1.85 | |
| | G/P | 4.26 | | **0.17** | |

**Fig. S3.**

**Carbon source dependent metabolic impacts on MCC and E/Q of WT Mtb and BCG**.

(**A**-**C**) Intrabacterial pool sizes of 2-methylcitrate (2MC) or 2-methylisocitrate (2MI) in WT Mtb or BCG cultured in media containing G/D carbons (**A**) and G/P carbons (**B**) are described as nmol/mg (normalized by co-extracted residual protein in the metabolome sample). Relative abundance of 2MC or 2MI in BCG to that of WT Mtb under G/P carbons (**C**).

(**D**-**F**) Intrabacterial pool sizes of glutamine (**D**) and glutamate (**E**) in WT Mtb or BCG cultured in media containing either G/D or G/P carbons are described as nmol/mg (normalized by co-extracted residual protein in the metabolome sample) and relative concentrations of glutamate to glutamine (E/Q ratio) in WT Mtb and BCG were calculated (**F**). *, P<0.001, not significant by Student's unpaired t-test.

(**G**) Summary of glutamine, glutamate and E/Q ratio in WT Mtb and BCG under varying carbon conditions. Red font indicates the lowest E/Q ratio value of BCG under G/P carbons.

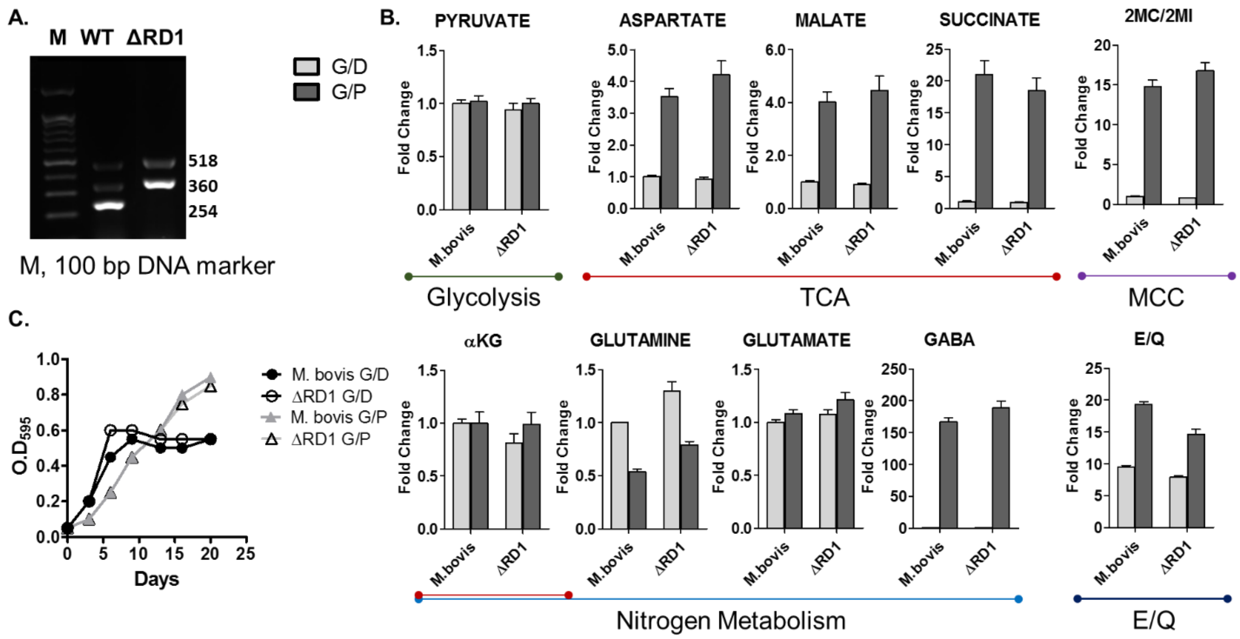


**Fig. S4.**

**Construction, validation, and characterization of RD1 deficient *M. bovis* (ΔRD1).**

(**A**) Validation of a ΔRD1 candidate by PCR using 3 sets of multiplex primers (RpoB-F and R, RD8-F and R, RD1-F and R) to detect *rpoB*, RD8 locus, and *esat6* genes. Primer sequences used to generate multiplex PCR products are listed in Table S1.

(**B**) Metabolomics profiles of WT *M. bovis* and ΔRD1. Focusing on the intermediates in the fatty acid metabolism (TCA cycle and MCC) and nitrogen metabolism (Q-E pathway and GABA shunt). All values are the average of two independent experiments, each of which consisted of biological triplicates ±s.e.m. αKG, α-ketoglutarate; 2MC, 2-methylcitrate; GABA, γ-aminobutyric acid; E/Q, glutamate/glutamine ratio.

(**C**) Growth phenotypes of WT *M. bovis* and ΔRD1 (reported by $O.D_{595}$) cultured in m7H9 media containing exogenous 40 mM sodium pyruvate and various carbon sources (glycerol, acetate, propionate, and dextrose) at 0.2% concentration for 20-day incubation. Growth phenotypes of WT *M. bovis* and ΔRD1 were monitored in m7H9 media with glycerol/dextrose (G/D) and glycerol/propionate (G/P) carbons. All values are the average of two independent experiments, each of which consisted of biological triplicates, ±SD.

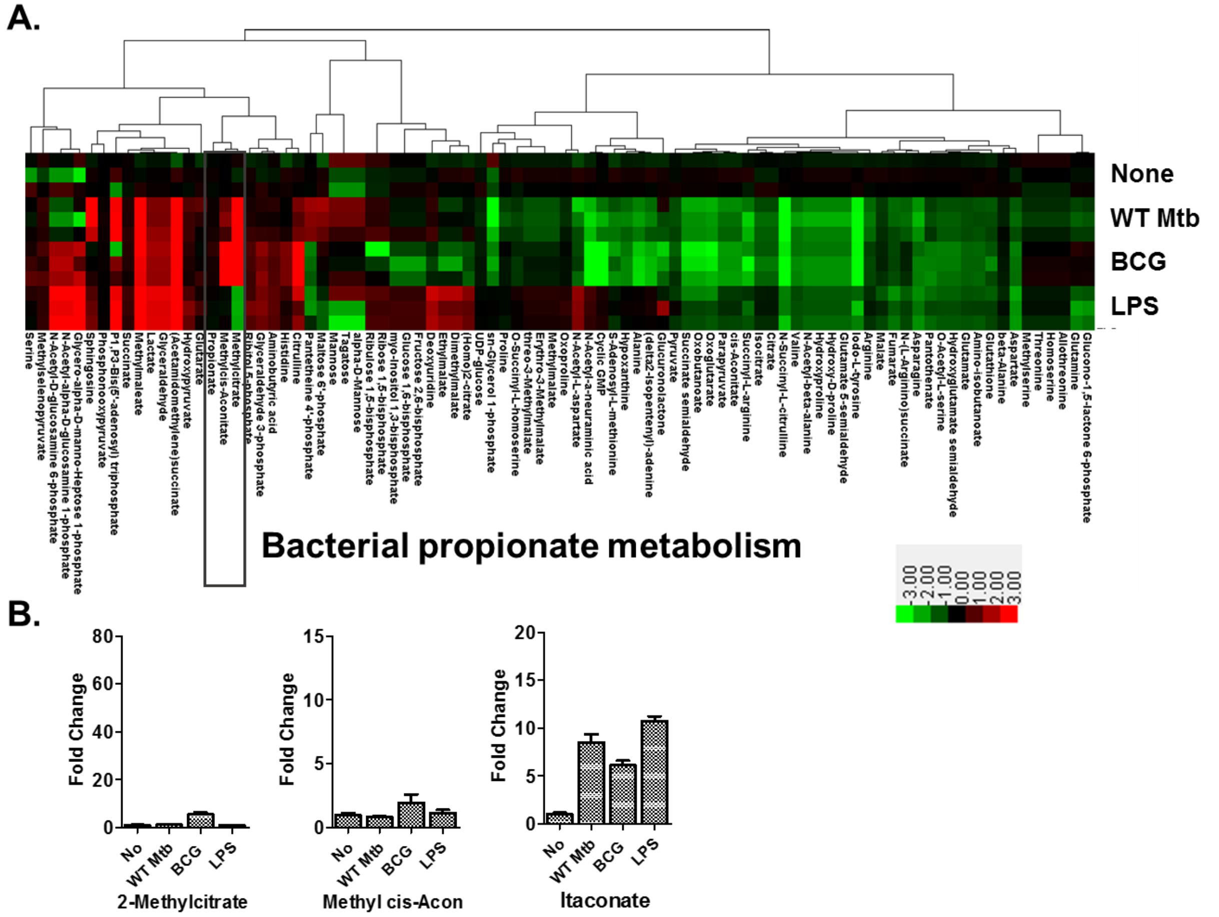


**Fig. S5.**

(**A**) Heatmap profile depicting levels of 95 detected metabolites extracted from THP-1 cells following either infection with WT Mtb or BCG at MOI 5.0, or treatment of 10 µg/mL LPS. Columns depict experimental conditions as indicated, starting with untreated controls followed by WT Mtb, BCG infection, and LPS treatment. Rows indicate individual metabolites. Data were parsed using uncentered Pearson’s correlation with centroid linkage clustering, and were rendered using the image generation programme treeview (http://jtreeview.sourceforge.net/). Data are depicted on a $\log_2$ scale relative to untreated control for each experimental condition.

(**B**) Relative abundance of chromatograms corresponding to 2-methylcitrate, 2-methyl cis-aconitate, and itaconate. The metabolome was extracted from THP-1 cells infected with either WT Mtb or BCG at MOI 5.0 and treated with LPS after removing un-lysed bacilli.

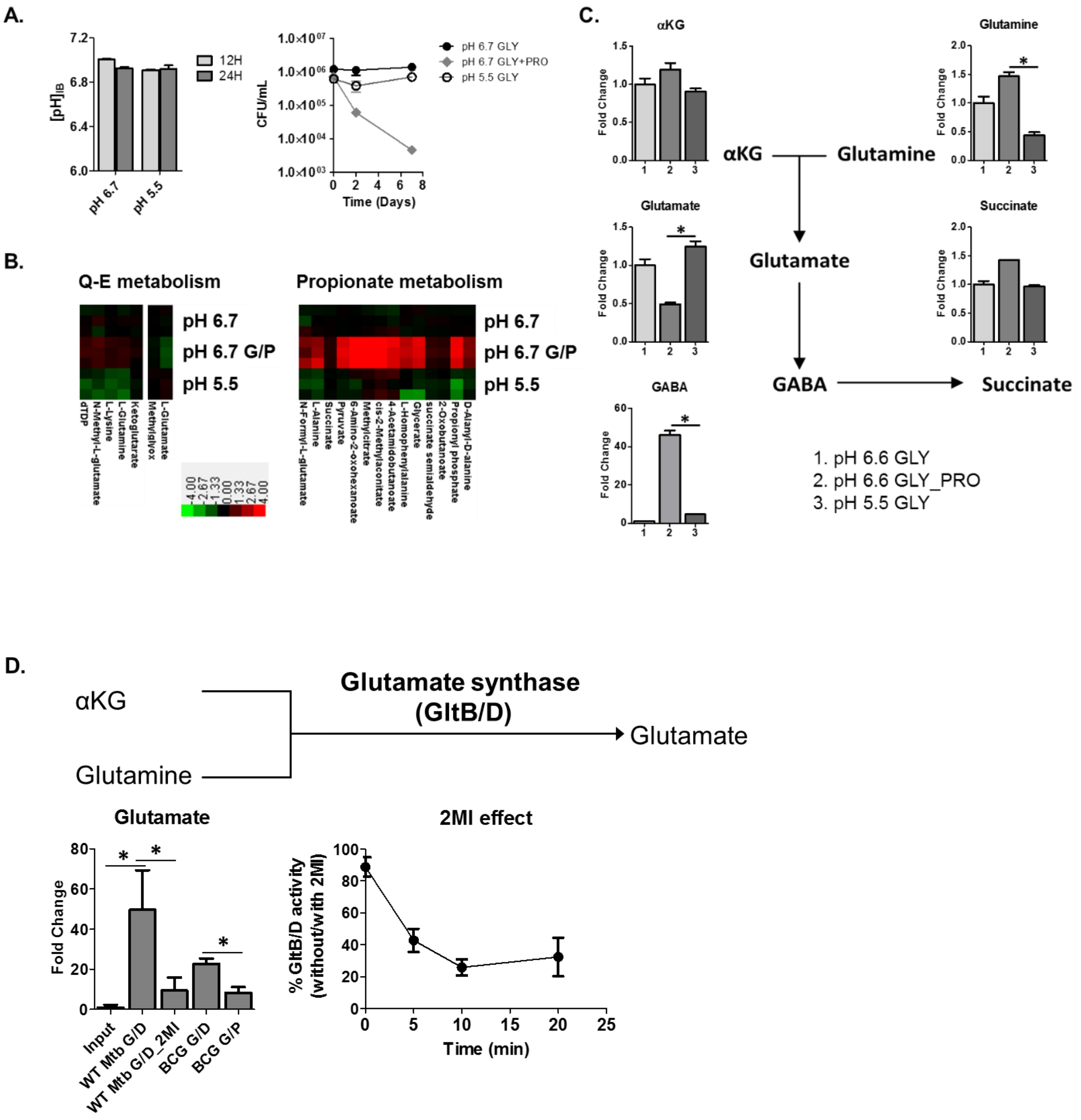
A.
7.2
6.8
6.4
6.0
[pH]IB
12H
24H
pH 6.7
pH 5.5
CFU/mL
Time (Days)
pH 6.7 GLY
pH 6.7 GLY+PRO
pH 5.5 GLY
B.
Q-E metabolism
Propionate metabolism
pH 6.7
pH 6.7 G/P
pH 5.5
dTDP
N-Methyl-L-glutamate
L-Lysine
L-Glutamine
Ketoglutarate
Methylglvox
L-Glutamate
N-Formyl-L-glutamate
L-Alanine
Succinate
Pyruvate
6-Amino-2-oxohexanoate
Methylcitrate
cis-2-Methylaconitate
4-Acetamidobutanoate
L-Homophenylalanine
Glycerate
succinate semialdehyde
2-Oxobutanoate
Propionyl phosphate
D-Alanyl-D-alanine
-4.00
-2.67
-1.33
0.00
1.33
2.67
4.00
C.
αKG
Glutamine
Glutamate
Succinate
GABA
Fold Change
αKG
Glutamine
Glutamate
GABA
Succinate
1. pH 6.6 GLY
2. pH 6.6 GLY_PRO
3. pH 5.5 GLY
D.
αKG
Glutamine
Glutamate synthase
(GltB/D)
Glutamate
Glutamate
Fold Change
Input
WT Mtb G/D
WT Mtb G/D_2MI
BCG G/D
BCG G/P
2MI effect
% GltB/D activity
(without/with 2MI)
Time (min)

**Fig. S6. Biochemical and metabolic comparison of BCG cultured under G/P carbons or acidic pH**. (**A**) Effect of culture conditions on intrabacterial pH and CFU-based viability of BCG cultured under acidic pH. (**B**) Heatmap profile depicting levels of intermediates in glutamine-glutamate metabolism of BCG under either G/P carbons or acidic pH (pH 5.5). Rows depict culture condition, starting with normal, G/P carbons, and acidic pH; columns indicate individual metabolites measured. Data were parsed as done in Fig. S5A. (**C**) Metabolomics profiles of BCG cultured under G/P carbons or acidic pH. Intrabacterial pool sizes of intermediates in nitrogen metabolism are depicted. Total bar heights indicate the relative intrabacterial pool sizes to counterpart in BCG cultured in glycerol single carbon.

(**D**) The effect of 2-methylisocitrate (2MI) on GltB/D mediated Q→E activity was monitored using an *in vitro* enzyme reaction. *In vitro* GltB/D reactions contained αKG (α-ketoglutarate) and glutamine as substrates and BCG or WT Mtb lysate (cultured under G/D or G/P carbons) in the presence of absence of 2MI. After 10 min incubation, the reaction was quenched by adding 100% acetonitrile with 0.2% formic acid and GltB/D activity was monitored by measuring glutamate production by LC-MS. 2MI inhibitory effect on WT Mtb GltB/D activity was also measured by the relative production (%) of glutamate in the absence or presence of 2MI in a time-dependent manner. All values are average of two independent experiments with biological triplicates, ±SD. *, $P<0.001$, not significant by Student's unpaired t-test.

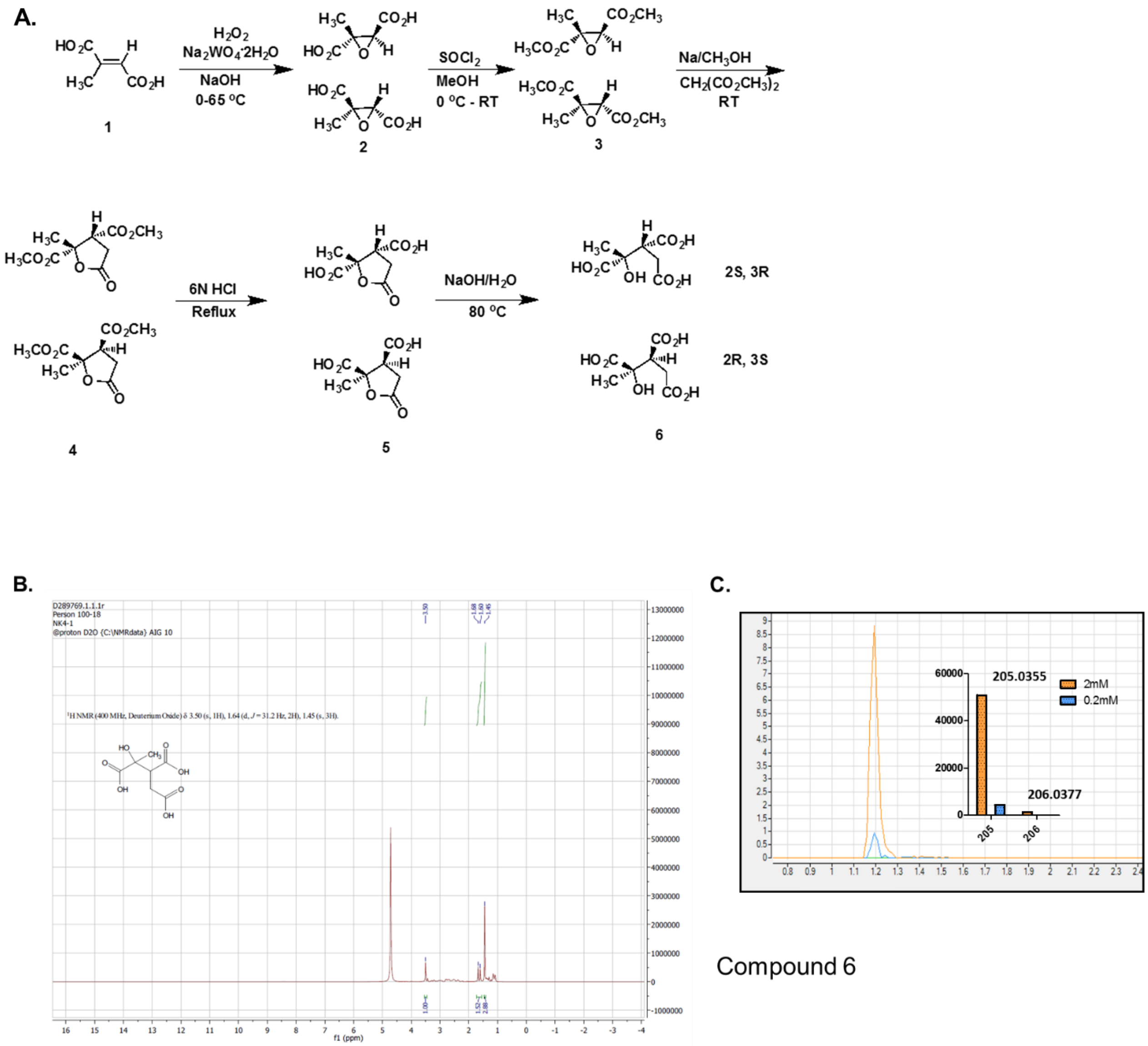


**Fig. S7. Chemical synthesis of D,L 2-methylisocitrate, ICL's MCL substrate in the MCC**. (**A**) Scheme of synthesis. *Reagents & Conditions:* 1→2. $H_2O$, $Na_2WO_4$-$2H_2O$, NaOH 0 – 65 $^oC$ for 8 hrs; 2→3. $SO_2Cl_2$, MeOH, 0 $^oC$ – RT, for 8 hrs; 3→4. Na/MeOH, $CH_2(CO_2CH_3)_2$, RT for 3 days; 4→5. 6 N, HCl, reflux, 0- 60 $^oC$, 3 h; 5→6. NaOH/$H_2O$, for 2 hrs. $^1$H-NMR of pure D,L 2-methylisocitrate (**B**) and LC-MS chromatogram of pure D,L 2-methylisocitrate at 0, 0.2, and 2 mM concentration (**C**).

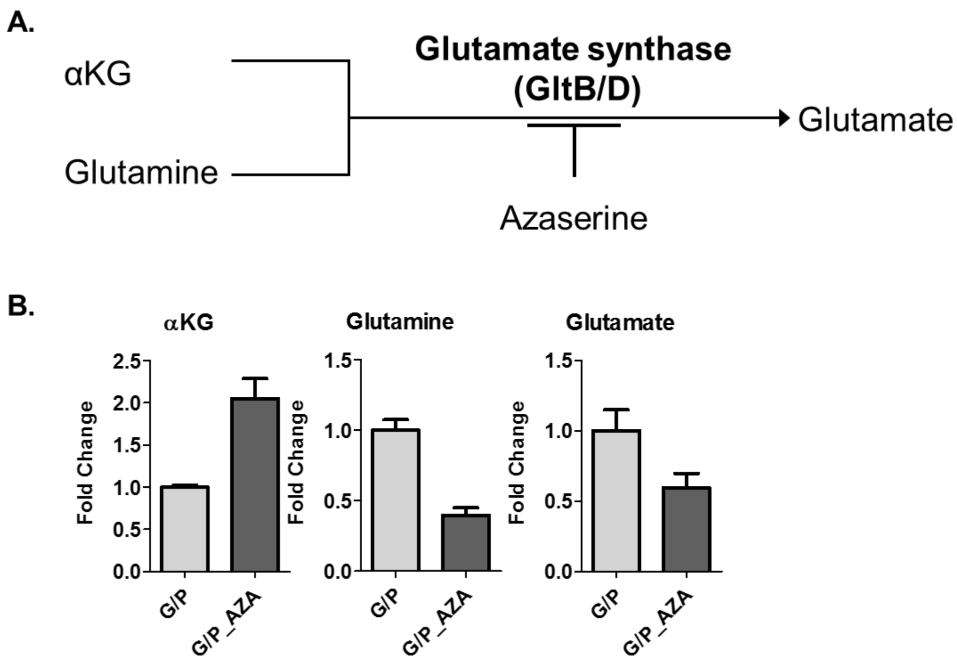


**Fig. S8. Metabolic effect of azaserine (AZA) on Mtb metabolism**. (**A**) Schematic describing the GltB/D (glutamate synthase) catalytic reaction in Mtb and a putative action of azaserine. (**B**) Metabolomics profile of GltB/D's substrates (αKG and glutamine) and product (glutamate) inside Mtb in the presence or absence of 1 μg/mL azaserine. *, P<0.001 by Student's unpaired t-test.

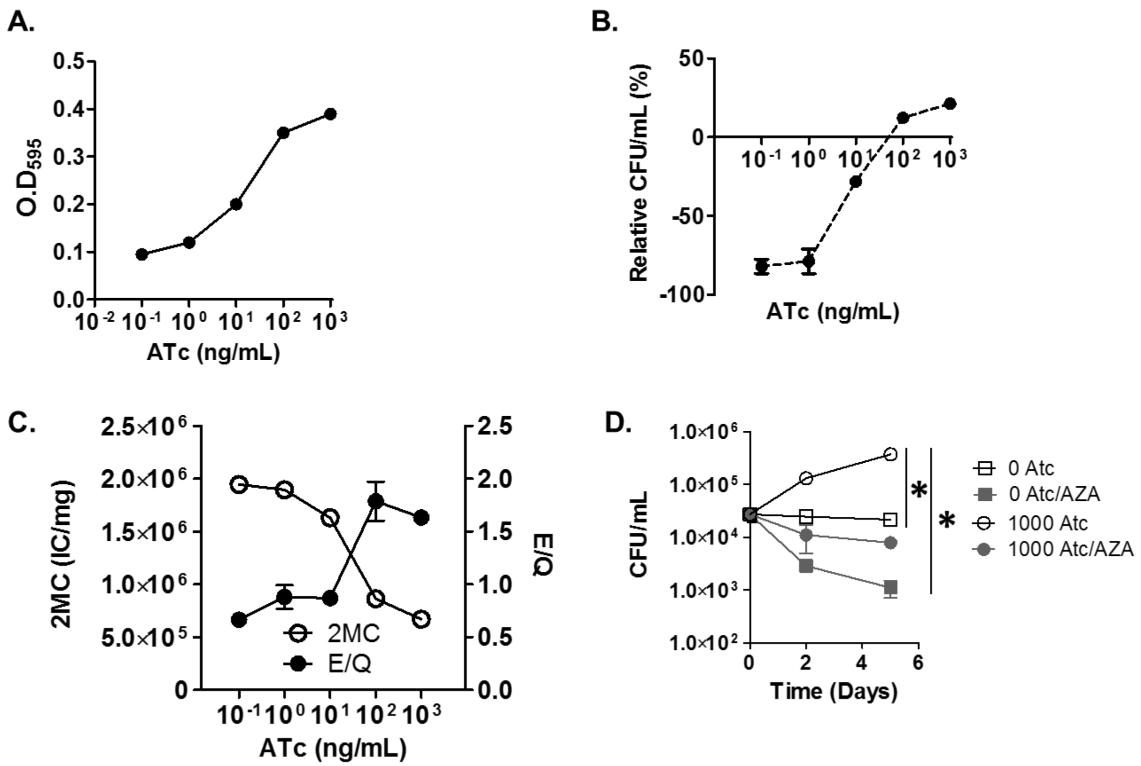


**Fig. S9. WT Mtb induces GltB/D mediated Q→E in catabolizing propionate carbon through MCC.** Functional role of crosstalk between MCC and Q-E conversion in Mtb during propionate assimilation. Five-day growth of ICL KD (**A**) or relative changes in CFU viability (**B**) in m7H9 with 0.2% glycerol and 0.1% propionate carbon sources with varying concentrations of anhydrotetracycline (ATc) ranging from 0 to 1000 ng/mL. (**C**) Correlation of levels of 2MC/2MI and E/Q rates in ICL KD at each ATc concentration when cultured in 0.2% glycerol and 0.1% propionate carbons. Absolute ion counts of 2MC/2MI are expressed as ion count/mg normalized by co-extracted protein content (y axis, left side). Pool sizes of Q and E are calculated using standard curves as done in Fig. S2 and the nmol/mg values are converted to E/Q rate (y axis, right side) over the ATc concentrations tested (x axis). (**D**) CFU-based viability of ICL KD to prove the synergistic bactericidal activity by co-inactivating ICL (MCL in MCC) and GltB/D (by 1 µg/mL Azaserine, AZA) cultured in the presence of either G/D or G/P carbons. *, P<0.001 by Student's unpaired t-test. All values are average of two independent experiments, each of which consisted of biological triplicates ± s.e.m.

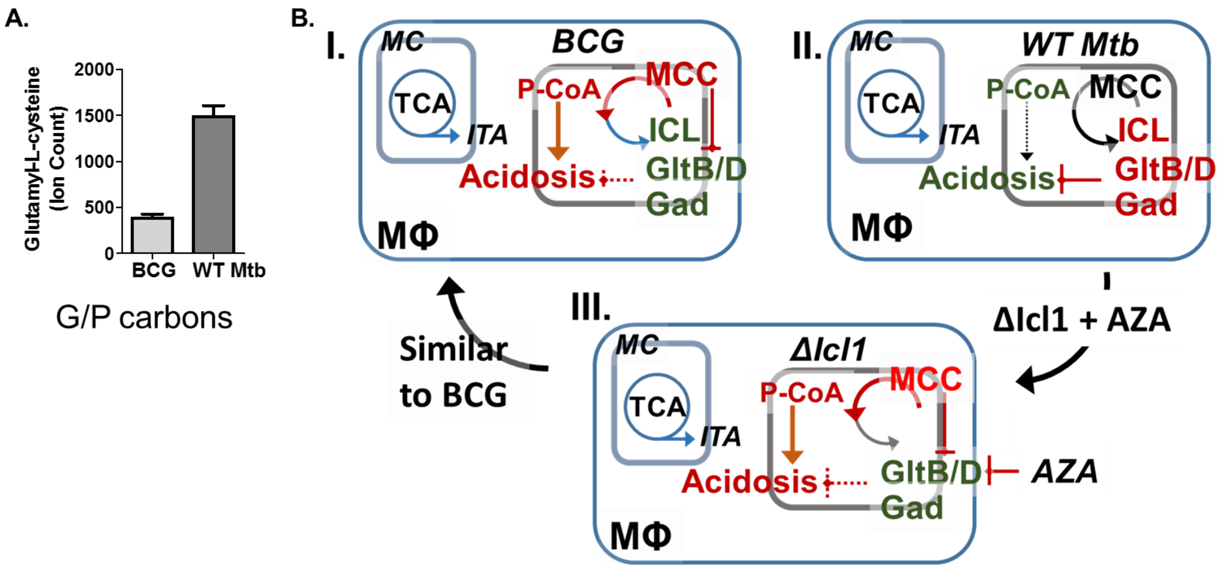


**Fig. S10. Metabolic neutralization to mitigate the propionate toxicity during overactive MCC: new drug target of Mtb.** (**A**) The metabolic effects of Q→E activity on the biosynthesis of glutamyl-L-cysteine, a precursor of ergothioneine. (**B**) **I.** Schematic describes the metabolic defect in adaptive metabolism of intracellular BCG that includes intrinsic downregulation of MCL (ICL) activity, subsequent accumulation of propionyl-CoA and MCC intermediates, and cytoplasmic acidification (acidosis). Acidosis is exacerbated by MCC-allosteric downregulation of Q→E activity (GltB/D) and the accompanied GABA shunt defect (Gad). **II.** In WT Mtb, unlike BCG, MCL activity is efficient in propionate assimilation, thereby maintaining MCC intermediates at lower levels. Propionate toxicity and associated cytoplasmic acidification were not observed because Q→E activity normally plays a role in neutralizing propionate toxicity. **III.** Crosstalk between Q→E and MCL is a potential drug target. Propionate toxicity in ICL deficient Mtb (ΔICL1) is drastically exacerbated by azaserine mediated GltB/D inhibition (co-inactivation of metabolic neutralization activity during propionate assimilation). As such, intracellular WT Mtb metabolism altered by inactivation of both ICL and GltB/D resembles that of BCG. Red letters or arrows indicate induced activities and green letters and arrows indicate decreased activities. MC, mitochondria; MΦ, macrophages; P-CoA, propionyl-CoA; ITA, itaconate; AZA, azaserine.

| Name | Sequence | Target | Expected size | |
|---|---|---|---|---|
| | | | WT | ΔRD1 |
| RpoB-F | GCT GGA CAT CTA CCG CAA GCT GC | *rpoB* | 518 | 518 |
| RpoB-R | CCG CGA GGC GAT CTG GCG GTT T | | | |
| RD8-F | GTC GAA GCG GGG CGC TCT | RD8 | 360 | 360 |
| RD8-R | GAA TTC CTG CTT CTC GCC CGA A | | | |
| RD1-F | CTG GCG GTC AAC CTG AAG AAG C | *esat6* | 254 | ND |
| RD1-R | AGG TCG AAC TCG CCC GAT CC | | | |

**Table S1.** Primer sets for multiplex PCR analysis as used in (Kim Y. *et al.* 2013)

| KEGG pathway | WT Mtb | | BCG | | LPS (up/down) |
|---|---|---|---|---|---|
| | up | down | up | down | |
| Starch and sucrose metabolism | 2 | 0 | 2 | 0 | 1/1 |
| Glycolysis / Gluconeogenesis | 4 | 0 | 4 | 0 | 4/0 |
| Pentose phosphate pathway | 3 | 2 | 4 | 1 | 3/2 |
| Fructose and mannose metabolism | 3 | 1 | 2 | 1 | 4/0 |
| Citrate cycle (TCA cycle) | 1 | 7 | 1 | 7 | 1/7 |
| Glyoxylate and dicarboxylate metabolism | 1 | 5 | 1 | 5 | 2/3 |
| C5-Branched dibasic acid metabolism | 1 | 6 | 1 | 4 | 4/3 |
| Propanoate metabolism | 3 | 2 | 3 | 2 | 0/5 |
| 2-Oxocarboxylic acid metabolism | 1 | 3 | 1 | 3 | 3/1 |
| Pyrimidine metabolism | 1 | 3 | 1 | 3 | 1/2 |
| Purine metabolism | 1 | 2 | 1 | 2 | 1/2 |
| Glycine, serine and threonine metabolism | 1 | 5 | 3 | 2 | 1/5 |
| Arginine/nitrogen biosynthesis | 1 | 8 | 3 | 5 | 1/3 |
| Valine, leucine and isoleucine degradation | 0 | 2 | 0 | 2 | 1/1 |
| Arginine and proline metabolism | 1 | 8 | 3 | 6 | 1/1 |
| Glycine, serine and threonine metabolism | 2 | 6 | 6 | 2 | 5/3 |
| Alanine, aspartate and glutamate metabolism | 2 | 10 | 2 | 10 | 6/4 |
| Pantothenate and CoA biosynthesis | 1 | 3 | 0 | 4 | 0/4 |
| β-alanine metabolism | 0 | 3 | 1 | 3 | 1/3 |
| Nicotinate and nicotinamide metabolism | 1 | 1 | 1 | 1 | 1/1 |

**Table S2.** Metabolic pathways of THP-1 cells altered by WT Mtb or BCG infection
